# Persistency of catastrophic out-of-pocket health expenditures: Measurement with evidence from three African countries - Malawi, Tanzania, and Uganda

**DOI:** 10.1016/j.socscimed.2024.117156

**Published:** 2024-09

**Authors:** Rocio Garcia-Diaz, Vishnu Prasad Sapkota, Gabriela Flores

**Affiliations:** a Tecnologico de Monterrey, School of Social Science and Government and Consultant to the World Health Organization, Geneva, Switzerland; b Department of Economics, Nepal Commerce Campus, Tribhuvan University, Nepal; c Consultant to the World Health Organization, Geneva, Switzerland; d Economic Evaluation and Analysis, Health Financing and Economics, World Health Organization, Avenua Appia 20, 1211, Geneva, Switzerland

## Abstract

Catastrophic out-of-pocket health expenditure (CHE) remain high in Sub-Saharan Africa and may not conform to the sporadic random pattern of acute illnesses that shapes insurance arrangements intended to avoid the risk of financial loss. The persistency of CHE remains a largely unexplored issue due to the lack of relevant methods and scarcity of panel data. This paper addresses the first shortcoming by presenting three different approaches to incorporating the timeframes into the analysis, considering dynamics between two periods, average over time and the recurrence of CHE incidence. Through the application of the complementary approaches, we identify (i) those at risk of persistent CHE in the short-term; (ii) those facing transient versus persistent CHE in the long-term; and (iii) those facing multiple CHE spells. The methods are applied to different definitions of CHE using panel data from three sub-Saharan countries: Malawi (3 waves: 2010, 2013, and 2016) with 4983 observations; Tanzania (3 waves: 2008, 2010, and 2012) with 8715 observations; and Uganda (5 waves: 2009, 2010, 2011, 2013, and 2015) with 6475 observations. All datasets are balanced panels. Additionally, we employ empirical strategies to identify the underlying factors contributing to these persistent and relatively high OOP. Across the three countries, we find that at least 27% of the people facing CHE in one period, because they spent more than 5% of their household budget on health out-of-pocket, will face it again in the next period. The lower-bound risk for those spending more than 10% of their household budget is 9% and for those spending more than 25% of their household capacity to pay is 13%. Between 11% and 45% of the population incurred CHE at least twice during the observation period when using the 5% budget definition of CHE. The double recurrence rate ranges between 7% and 13% when using the 25% capacity-to-pay definition and between 3% and 20% when using the 10% budgetshare definition. Between 22% and 32% of the population experienced chronic CHE at the 5% of the budgetshare definition (6%–10% at the 10% of the budgetshare definition of CHE; 2%–11% at 25% of capacity-to-pay). Our panel regression analysis consistently highlights the susceptibility of certain groups to face persistence CHE, notably those residing in rural areas, individuals with lower levels of education, the elderly, and those who have undergone hospitalizations.

## Highlights

• We propose to assess persistent catastrophic health expenses via risk exposure, average changes and recurrence.
• At least 27% facing catastrophic health expenses in Malawi, Uganda, and Tanzania will face it again.
• Elderly, female heads, and health shocks increase catastrophic health expense persistence.
• Longitudinal insights identify enduring financial vulnerabilities due to out-of-pocket health spending.

## 1 Introduction

Out-of-pocket health payments (OOP) are a predominant form of healthcare financing in Sub-Saharan Africa (WHO, 2024). In 2021, 24 out of the 49 countries in that region funded over 30% of their total current health spending through OOP, slightly less than in 2019 (Ifeagwu et al., 2021). While the health financing perspective is important as it emphasizes to what extent the health system relies on people's direct contributions (OOPs), a separate tracking is needed to understand the implications of such contributions on people's living standards and ability to meet other needs. This understanding is crucial for assessing how these burdens compromise the attainment of the sustainable development goals (SDGs) (WHO, 2019).

Catastrophic health expenditure, hereafter (CHE) is a key metric for monitoring this impact. It is characterized by OOP surpassing 10% of total household consumption, expenditure, or income, hereafter called the budget share approach, which is the one adopted for the SDGs (SDG indicator 3.8.2) (WHO, 2023). Other definitions exist, such as OOP exceeding either 40% of non-food expenditures(Wagstaff and Doorslaer, 2003), 40% of non-essential spending on food (Xu et al., 2003), or 40% of non-essential spending on food, housing, and utilities(Cylus et al., 2018), hereafter called the capacity-to-pay approach. However, all these conventional metrics are usually assessed at a single time point, failing to identify if CHE persistently affects the same people. This paper proposes three methods to assess persistent CHE using panel data from three Sub-Saharan African countries.

In Sub-Saharan Africa, OOP exceeding 10% of household consumption affected 98.7 million people in 2019 (8.8% of the population), increasing on average at 2.6 million people per year since 2000 (WHO, 2023). Moreover, 111 publications covering a total of 1,040,620 households across 31 sub-Saharan countries found a pooled annual incidence of 8.7% of households spending over 40% of their non-food expenditure on health out of pocket, thereby forcing households to compromise other basic needs, liquidate assets or accumulate debt (Eze et al., 2022). In the three countries analyzed in this paper, OOP exceeding 10% of household's budget respectively affected 3%, 4.3%, and 15.3% of the populations in Malawi in 2019, Tanzania in 2018, and Uganda in 2016, up from 0.3% (Malawi-2004), 5% (Tanzania-2000) and 9.4% (Uganda, 1999; WHO, 2023).

This enduring pattern of CHE stems from a lack of financial protection when households access healthcare and they are required to pay for the services they need, when they need them, exacerbating the economic burden on families during illnesses. Particularly, chronic non-communicable diseases such as cardiovascular diseases, cancer, chronic respiratory diseases, and diabetes are increasingly adding to the global burden of diseases, especially in low- and middle-income countries (Abegunde et al., 2007; Murphy et al., 2020; Yach et al., 2005). Since chronic diseases require long-term treatment, the resulting financial strain tends to be more severe compared to acute illnesses (Odunyemi et al., 2023; Olmen et al., 2011).

Moreover, specific populations face heightened challenges. For instance, households affected by HIV/AIDS in Nigeria, experience the highest CHE, with 100% spending above 10% of the budget share and 94.3% spending more than 40% of their capacity-to-pay (Adisa, 2015). Similarly, hospitalized children with Malaria in the Democratic Republic of Congo face substantial financial burdens, with CHE reaching 81.1% of the capacity-to-pay, based on the 40% threshold (Ilunga-Ilunga et al., 2015). Even when healthcare is reported as free, surgical care represents a significant proportion of household total consumption, ranging from 60% to 90% (Okoroh and Riviello, 2021).

This paper is focused on CHE given the evidence that it has been increasing continuously over the past twenty years everywhere (WHOWorld Bank, 2023). However, to date, to the best of our knowledge, no method has been proposed to assess the extent to which these type of expenditure affects the same individual.

The consequences of health events straining household expenditure are often studied through consumption smoothing following a health shock. Studies typically focus on fluctuations in consumption due to a single health episode (Dercon, 2002; Gertler and Gruber, 2002; Onisanwa and Olaniyan, 2019). However, chronic, or long-term illnesses often involve multiple health episodes leading to reduced consumption of both food and non-food items (Onisanwa and Olaniyan, 2019; Wang et al., 2023), longer treatment duration with associated direct and indirect costs (Damme et al., 2004; Russell, 2004) and, eventually, a decline in permanent labor income (Shrinivas et al., 2023).

The impact of illness events varies by duration and severity (Njagi et al., 2018; Zhao et al., 2020). These events often represent continuous health deterioration linked to OOP (Njagi et al., 2018; Russell, 2004). Consequently, a challenge in the literature is classifying health shocks by duration and severity, along with the resulting financial hardship.

Panel data analysis has been a valuable tool for assessing health coverage for poverty-caused illness (Wang et al., 2023), health insurance impact (Fattah et al., 2023; Kanmiki et al., 2019), or financial hardship due to OOP. But in the latter, no study attempted to investigate persistency (Rahman et al., 2022). Some studies focused on trends over time within countries using time series analysis, such as (Haakenstad et al., 2023) which again fails to uncover whether CHE is a short or long-term phenomenon.

We propose to apply three distinct approaches to evaluate the persistence of conventional indicators of CHE: (i) The transitional probabilities approach, frequently used in the poverty indicator litterature (Baulch and Masset, 2003; Radeny et al., 2012; Woolard and Klasen, 2005), involves an assessment of conditional probabilities of transitioning between different states across adjacent waves. (ii) The mean approach to identify stages of persistent or transient poverty developed by (Jalan and Ravallion, 2000) that we suggest applying to CHE by comparing for each household, the occurrence of CHE at a given point in time to their time mean CHE. The latter is based on the average ratio of household's OOP spending over household's resources exceeding a given threshold. (iii) The last method follows the counting approach for chronic poverty proposed by (Foster, 2007; Foster and Santos, 2012) that can be used to identify the degree of recurrence of CHE. To this end we employ a time threshold duration to differentiate recurrent CHE.

The three proposed approaches are complementary and respectively capture the risk of persistency of a given state (CHE or no CHE) between 2 periods; persistence over time; and the frequency of CHE. However, they differ in their applicability which depends on two key aspects: the number of periods and which persistency characteristic is of interest as further explained in the methods section. However, the three approaches have a common objective: they aim to broaden the analysis of financial protection to multiple time periods for better evidence-based policy discussions. For instance, while two countries may exhibit the same incidence of CHE at a particular moment, the nature of this burden may differ. In one country, the same individuals might face such spending. While in the other, it could be different individuals each time. Consequently, the appropriate policy response may vary; targeted interventions might be necessary in the former, while improved risk pooling might suffice in the latter.

In addition to characterizing the persistency of CHE, we use empirical regression analysis to uncover the underlying factors driving the observed CHE outcomes, utilizing pooled ordinary least squares (OLS) regression analysis.

The paper is structured as follows: Section 2 details the methods for assessing CHE over time, Section 3 describes the data and variables, Section 4 presents the results, and Section 5 provides discussions and conclusions.

## 2 Materials and methods

### 2.1 Methods

**Fig. 1:**
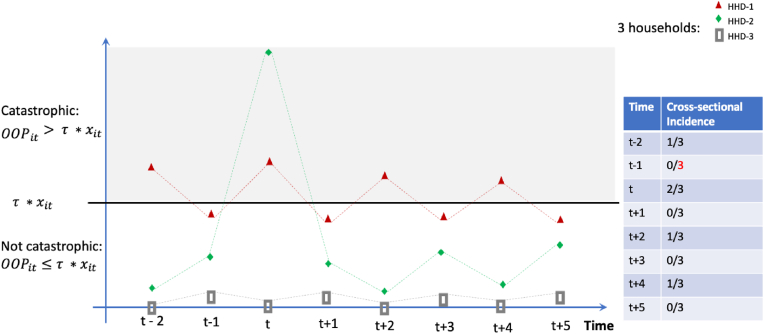
Household's Out-of-pocket health spending (OOP).

OOP (${OOP}_{it}$) refers to the payment made by households i at a given point t. It is considered catastrophic (CHE) when it exceeds a given fraction τ of household's resources available to pay for health care, $x_{it}$, (${\text{OOP}}_{it}>\tau *x_{it})$ , which may lead to cutting consumption of other needs. In the standard static cross-sectional approach, the analyst can only monitor the prevalence of CHE for different samples over time. When panel data is available, the same sample is observed over time which opens the possibility to characterize the transient or persistent nature of CHE. To date, to the best of our knowledge, no one has proposed any method to do so despite the growing availability of panels and the rapid development of high-frequency surveys. Fig. 1 aims to explain intuitively why it is important to adopt other approaches to analyze panel data, in which case each one of the three proposed methods in this paper can be used and how they complement each other.

Let's assume there are only 3 households in a country. They have the same household size, and the same resources available to pay for health services and products but their OOP profile is different (Fig. 1). Let's also assume that the data analyst has access to the panel of this imaginary country with 3 households but initially just for 2 periods. If these two periods are “t” and “t+2”, the cross-sectional incidence of CHE drops from 66% (or 2 out of 3 households) to 33% (or 1 of 3 households). But without further analysis, it is impossible to know if those facing CHE in period t+2 are a subset of those incurring such spending in period “t” or other ones. We first propose to use the transitional probabilities approach widely used to explore poverty dynamics.

(Radeny et al., 2012; Woolard and Klasen, 2005). The poverty literature underlines that there is a substantial flow of households into and out of poverty, even when the published statistics remain the same, decrease, or increase (Radeny et al., 2012). In the context of CHE, there is a similar concern. Similarly, in the context of CHE, this method addresses concerns about persistency. Using two-time points, it calculates conditional probability changes from one CHE status at a baseline period to another in the subsequent period, providing insights into persistency risks and the sustainability of household coping strategies over time.

However, the transitional probability approach is restricted to pairwise comparisons, potentially underestimating persistency risks over multiple periods. Moreover, it relies on an uncertain baseline selection (Foucrier et al., 2022). For example, if the true baseline is at t-2, starting at time t may overestimate the number of households transitioning out of CHE. Therefore, additional methods may be necessary when more than two time periods are considered.

The mean approach was first proposed by (Jalan and Ravallion, 2000) to differentiate transient from chronic poverty. Drawing on their work, we propose to define the persistent component of an intertemporal indicator of CHE as the average OOP exceeding a given fraction of the average resources available to pay for health care. The threshold τ is a given value of household resources. In other words, consider as if neither the level of those resources varied over time nor the level of OOP health spending. For simplicity, in our imaginary country depicted in Fig. 1, that level is also assumed to be equal for all 3 households ($x_{it}\equiv x_{i})$. But among these three households, only the first and second ones has an average level of OOP exceeding the constant level of resources available to pay for health illustrated by the flat line at $\tau *x_{it}$. The first one because OOP exceeds the flat line every two periods, so its mean will also be above the flat line. For the second one, OOP exceeds ability-to-pay only once but please assume that the increase was so high that its average OOP is also above the flat line. This assumption is made to illustrate the point that an acute payment made by a household during a single period would be deemed to be persistent in the mean approach, if it significantly affects the overall mean, thereby influencing the capacity-to-pay over subsequent periods. The other approaches would not capture the persistency of a single OOP at all. The other household in this example does not have an average OOP exceeding the average ability to pay. CHE is only transient using the mean approach but there are other notable differences between the 3 households in this imaginary country. The third one never face catastrophic OOP health spending because nothing or very little is spent on health out of pocket. To capture the differences between these 3 last households, we propose to use the counting approach.

The CHE for the household i at a time t, is measured by $C{HE}_{it}\left(OOP_{it}/x_{it};\tau \right)$ is 1 when $OOP_{it}>\tau {*x}_{it}$ , and 0, otherwise. In this intertemporal set-up, the proportion τ is fixed across all periods.

#### 2.1.1 Transitional probabilities

Transition probabilities are computed between two points in time, t=1,2. Given the CHE in the initial period, $C{HE}_{i1}\left(OOP_{i1}/x_{i1};\tau \right)$, we calculate the conditional probability of changes from one status to another in the second period $C{HE}_{i2}\left(OOP_{i2}/x_{i2};\tau \right)$ to understand the risk of persistency without controlling for the households’ characteristics. The joint probability of incurring CHE in both periods is the incidence in the second period (CHE_t=2_) multiplied by the conditional probability of CHE in the second period conditional on facing CHE in the first period (Prob($C{HE}_{i2}==1|C{HE}_{i1}==1)$).

For more than two periods, estimating joint probabilities requires calculating all possible combinations (e.g., periods 1 and 3; 1 and 4, etc.) and the incidence in all periods, best represented by a matrix. This estimation helps understand CHE dynamics (entry or exit). If the focus is on the number of CHE occurrences (e.g., twice, thrice), the counting approach is simpler, as explained later. With multiple periods, the mean approach identifies transient and persistent components of CHE using the time-series properties of OOP ratios around its mean.

#### 2.1.2 The mean approach

The mean approach considers a panel data model that incorporates information from more than two points in time. Following (Jalan and Ravallion, 2000) a household i may experience financial stress due to OOP health spending across different periods of time give threshold, τ that is, ${CHE}_{it}\left({OOP}_{it}/x_{it};\tau \right)$ for all t=1,⋯,T.

The chronic component of CHE is identified by,

$${CR}_{i}=\bar{C{HE}_{i}}\left[\bar{\left(\frac{OOP_{i}}{x_{i}}\right)};\tau \right]$$

where time-mean CHE for household i, $\bar{C{HE}_{i}}\left[\bar{\left(\frac{OOP_{i}}{x_{i}}\right)};\tau \right]$ is 1 when $\bar{\left(\frac{OOP_{i}}{x_{i}}\right)}>\tau$ and 0 otherwise, with $\bar{\left(\frac{{OOP}_{i}}{x_{i}}\right)}=\frac{1}{T}\sum_{t=1}^{T}\left({OOP}_{it}/x_{it}\right)$.

It can be further decomposed into four types of populations: (i) Those that face CHE at all periods with ${CR}_{i}=1$ and $C{HE}_{i}=1\;\text{because}\;C{HE}_{it}=1\;\forall t$ ; (ii) Those that face persistent CHE, that is, have a mean-time OOP share exceeding the threshold over the period of analysis but have at least one period with no CHE (${CR}_{i}=1\;but\;C{HE}_{it}\neq 1\;\forall t\;)$; (iii) Those with transient CHE (${TR}_{i}\neq 0\;and\;{CR}_{i}=0)$, they occasionally face CHE but their mean-time OOP share does not exceed the threshold during the period of the analysis; and (iv) Those that never experience CHE in the period of analysis as their mean-time OOP share is always below the threshold and so is their OOP share in every period (${TR}_{i}={CR}_{i}=C{HE}_{i}=C{HE}_{it}=0)$.

The overall intertemporal CHE corresponds to the weighted sum of the transient and chronic components. The weights may correspond to the household weight multiplied by the household size to obtain representative estimates at the household level or simply to the household weight if the unit of analysis of interest is the household.

#### 2.1.3 The counting approach

The counting approach identifies households experiencing CHE over time using an identification duration criterion proposed by Foster and Santos (2012). According to this criterion, a household is considered to have chronic CHE if it consistently faces k periods with such outcomes. First, for a given period *T*, at the household level, we identify all CHE spells. This is denoted by $C{HE}_{i}.=\left(\begin{array}{cccc} C{HE}_{i1}, & C{HE}_{i2}, & \cdots , & C{HE}_{iT} \end{array}\right)\text{.}$ Second, still, at the household level, we count the number of periods a household i faced CHE outcomes for a given threshold τ, as $c_{i}=\sum_{t=1}^{T}C{HE}_{it}\left({OOP}_{it}/x_{it};\tau \right)$ . Third, we apply the duration threshold “k” and identify the chronic occurrence (or counting) with $c_{i}\geq k$ by which is equal to 1 when a household faces chronic CHE, according to the proportion threshold, τ, and the duration identification cut-off, k and 0 otherwise. Lastly, we take the weighted mean of $c_{i}$ across all households to identify the chronic occurrence in the population denoted $C^{c}\left(\tau ;k\right)$.

The counting approach focuses on recurrence (Atkinson, 2003), whereas CHE only captures incidence (Seth, 2013). Thus, if a household incurs a substantial OOP in one period but not in subsequent periods, the counting approach will not classify it as recurrent (Seth, 2013). This method may overlook the lasting impacts of health shocks on a household's medium- and long-term capacity-to-pay. However, the counting period allows for assessing the chronic financial stress households face due to repeated OOP, which may deplete coping mechanisms.

The three approaches offer distinct perspectives on the persistence of CHE over time. Transitional probabilities focus on pairwise conditional risks, tracking changes in status from one point to another. The mean approach provides insights into the variability versus stability of CHE, examining how OOP influences the average allocation of resources and capacity to pay overtime. The counting approach highlights the chronicity of occurrences, requiring a parameter to identify duration or chronicity. While the mean approach centers on resource allocation following an OOP event, the counting approach emphasizes repeated financial stress due to recurrent OOP.

In this study, we illustrate the application of the three proposed approaches using the first two definitions of CHE proposed in 2003 by (Wagstaff and Doorslaer, 2003) and (Xu et al., 2003) respectively. The first one compares a household's OOP to the household total budget defined as household consumption comprising the monetary value of all goods and services as well as home-made products. We use the 10% threshold adopted to identify CHE within the SDG monitoring framework. But we also used a lower threshold of 5% of household total consumption to better capture the persistency of OOP among the poorest. Indeed, latest evidence for low-income countries has shown that even small amounts below 10% of the household total budget are a source of financial hardship, especially for the poorest (WHOWorld Bank, 2023). Only 9–13% of the population incurring financial hardship due to OOP is pushed or further pushed into poverty by OOP exceeding 10% (WHOWorld Bank, 2023). Moreover, in general, the incidence at the 10% threshold in the Sub-Saharan region fluctuates between 8 and 9.5% since 2000 (WHOWorld Bank, 2023). Therefore, using a lower threshold of 5% allows us to have enough observations to test the three approaches in the empirical application and capture the financial hardship experienced by poorer people.

The second definition proposed in 2003 compares a household's OOP health spending to a household's total consumption net of actual spending on food (Wagstaff and Doorslaer, 2003) or basic spending on food (Xu et al., 2003).To reduce measurement error, the latter corresponds to the average food expenditures of households whose food budget share falls between the 45th and 55th percentile of the entire sample. With this measure of household resources available to pay for health care, we use a 25% threshold to identify CHE based on non-basic food consumption. Finally, the incidence of CHE is sometimes reported in terms of the proportion of the population like in SDG indicator 3.8.2 and sometimes in terms of the share of households. We use population as our unit of analysis for the empirical study.

### 2.2 Empirical strategy to identify the drivers of persistence

To identify the driving factors of persistent CHE within households, we use a pooled OLS regression model (1), where the dependent variables encompass the three specifications: (i) the first difference of CHE dummies, (ii) the mean-based chronicity measures (including transient and persistence outcomes) and (iii) the counting-based chronicity measures over a specified number of periods (k). These model specifications will enable us to comprehensively explore and analyze the factors driving the persistence of CHE.

(1) $$y_{it}=a+x_{it}^{\prime }\beta +\varepsilon_{it}$$

where the error term follows a normal distribution.

For the transitional matrices, the dependent variable $y_{it}$ reflects the change in status in terms of CHE from period 1, $C{HE}_{i1}\left(OOP_{i1}/x_{i1};\tau \right)$, to period 2, $C{HE}_{i2}\left(OOP_{i2}/x_{i2};\tau \right)$ where it takes value of 1 if household move into CHE in period 2, 0 if there is no change in CHE state and -1 when the households move out of CHE in period 2.

When employing the mean approach, we categorize households as either facing persistent OOP health spending (PERSISTENT) or dealing with transient OOP health spending (TRANSIENT). Conversely, when employing the counting approach, we identify households with chronic CHE based on predefined k periods threshold (CHRONIC, k).

Using different measures of persistence in OOP health spending, we can assess if their determinants vary or if there are common factors. The insights from this analysis help improve healthcare financing and mechanisms, enhancing well-being and financial security in households.

## 3 Data

We use data from nationally representative surveys in Malawi, Tanzania, and Uganda. For Uganda, we use data from the Uganda National Panel Survey (UNPS) waves 1 (2009–2010), 2 (2010–2011), 3 (2011–2012), 4 (2013–2014), and 5 (2015–2016). For Malawi, we use data from the Malawi Integrated Household Survey (IHPS) waves 1 (2010–2011), 2 (2013–2014), and 3 (2016–2017). For Tanzania, we use data from the Tanzania National Panel Survey (TZNPS) waves 1 (2008–2009), 2 (2010–2011), and 3 (2012–2013). The surveys were selected based on panel data availability, forming a balanced panel of households appearing in each survey wave with variables such as urban/rural residence, income or expenditures, and indicators covering household members' health, education, and infrastructure characteristics. Balancing the sample into a panel of equal representation is challenging, requiring a tradeoff between the number of households observed and the tracking duration. Our goal is to measure the persistence of CHE. To illustrate different approaches, we monitor the same households across survey waves to ensure our aggregated estimates reflect longitudinal trends accurately, avoiding biases from household entry and exit. Comparing country panels is challenging due to variations in panel lengths. To address this, we base our measure of CHE persistence on the average taken over waves, which alleviates potential bias from panel length variations. The panel structures and methods for handling attrition are detailed in Supplementary Material, Table 1.

### 3.1 Variables

To be able to compare across time, our welfare variables related to expenditure have been normalized for differences in demographics and prices. We use annual per capita expenditure and deflate our data using 2011 as a base year for changes in prices during the period of analysis.

Total OOP expenditures encompass all direct healthcare costs incurred during treatment, including medicines, physician/consultation fees, health provider charges, and diagnostics for inpatient care. OOP for medicines specifically covers outpatient medication costs. Notably, our definition excludes non-medical expenses such as transportation and lodging, aligning with standard OOP definitions (WHO, 2019).

Our comprehensive analysis of determinants draws on a range of variables from literature on health financial protection indicators (Kanmiki et al., 2019), incorporating cross-sectional (Njagi et al., 2018) and panel data analyses (Fattah et al., 2023; Li et al., 2023). Household head characteristics, including age, gender, education level, marital status, and labor force participation, are considered. Labor force status is expected not only to increase income and reduce financial risk but also to enhance the likelihood of household members having work-related insurance.

Additionally, household characteristics such as size and composition (including children under 5 and older adults), household and land ownership status, health shocks, hospitalizations, and healthcare-seeking behavior are included where data permit. Unfortunately, we are unable to identify chronic illnesses within households, which we consider an important factor influencing persistence. In this respect, age composition serves as our closest proxy for this factor. We also incorporate a dummy variable indicating rural or urban residence. In the Supplementary Material Table 3, we provide sample estimates for individual years. All analyses were conducted using STATA version 15.

Table 1 shows the pooled statistics of the variables covered in the analysis. OOP stands around 644 for Uganda, 629 for Tanzania and 36 for Malawi, expressed in per capita per annum terms in local currency units at 2011 prices. In the Supplementary Material, Table 3, we show the yearly trend of the OOP spending. In Table 1, row 2 to 4, we present estimates of CHE using different definitions. Between one in four and one in five people face CHE at 5% of budget share at some point in time. Between 9% (Tanzania) and 12% (Uganda and Malawi) incur CHE using the 10% threshold. On the other hand, the corresponding figure using the 25% threshold of capacity-to-pay are lower for Uganda (8.1%) and higher for Tanzania (14.7%) and Malawi (15.7%). Regarding the geographic and demographic composition of the households, the majority of households are rural, most also own their household in Uganda and Tanzania, and the pooled average household size ranges between 5.5 in Malawi to 6.9 in Tanzania. Most heads are male, with a primary level of education, married and monogamous in Uganda and Tanzania; working on average 26 h per week in Uganda versus 7.5 in Malawi. Relatively higher percentage of households have used hospital services in Tanzania (38%) followed by Uganda (14.2%).

**Table 1:** Sample statistics of the variables used in the analysis.

Variables	Uganda		Tanzania		Malawi	
	Mean	Std. Err.	Mean	Std. Err.	Mean	Std. Err.
**A. OOP spending and CHE incidence**						
OOP spending per capita annually	644.4	3008.7	629.3	5185.6	35.7	118.1
CHE across annual per-capita household expenditure, 5% threshold	24.3%	0.7%	20.7%	0.6%	22.7%	0.8%
CHE across annual per-capita household expenditure, 10% threshold	11.8%	0.5%	8.7%	0.4%	11.9%	0.6%
CHE using Capacity to pay at 25% of annual per-capita household expenditure	8.1%	0.4%	14.7%	0.5%	15.4%	0.7%
**B. Health utilization/shocks**						
Have used hospital care	0.1422	0.3493	0.3779	0.7352	0.0915	0.2883
Have used outpatient care	0.0198	0.1394	0.7814	1.0864	0.2020	0.4015
Household head health shocks [%]	42.5%	49.4%	8.6%	28.1%	22.1%	41.5%
**C. Household demographics**						
Age of the household head is over 60 years [%]	18.5%	38.8%	20.2%	40.2%	17.6%	38.1%
Household head is female [%]	39.8%	49.0%	20.4%	40.3%	26.7%	44.2%
**Education of household head**						
No education [%]	22.2%	41.6%	21.9%	41.3%	97.0%	17.0%
Primary education [%]	53.0%	49.9%	68.0%	46.6%	3.0%	17.0%
Secondary education [%]	17.0%	37.5%	9.7%	29.6%	0.0%	–
Higher education [%]	7.8%	26.9%	0.4%	6.0%	0.0%	–
**Marital status of the household head**						
Monogamous [%]	50.7%	50.0%	67.5%	46.8%	1.5%	12.2%
Polygamous [%]	19.3%	39.4%	13.9%	34.6%	1.0%	10.1%
Divorced/separated [%]	11.4%	31.8%	6.3%	24.3%	79.8%	40.2%
Widower [%]	15.9%	36.5%	10.1%	30.2%	16.8%	37.4%
Never married [%]	2.8%	16.4%	2.2%	14.6%	0.9%	9.4%
**Employment situation household head**						
Hours worked per week	25.8	22.5	–	–	7.5	20.2
Employment status [%]	–	–	–	–	31.5%	46.4%
**Members age in years**						
Children between 0 and 5	1.8316	1.5655	2.1427	1.7352	1.8408	1.4016
Household members aged 60 or more	0.8603	1.4348	0.3478	0.6072	0.2757	0.5701
**Household head characteristics**						
Urban [%]	18.9%	39.1%	24.2%	42.8%	16.5%	37.1%
Household owned [%]	64.6%	47.8%	85.5%	35.2%	–	–
Household size	6.5	3.2	6.9	3.4	5.5	2.8
**Number of households [H]**	**1295**		**2905**		**1661**	
**Number of waves [T]**	**5**		**3**		**3**	
**Number of observations [H x T]**	**6475**		**8715**		**4983**	

**Note:** The reported statistics are based on balanced panel using longitudinal Iweights pooled over the respective number of waves. Malawi IHPS for years 2010, 2013 and 2016; Tanzania TNSP for years 2008, 2010 and 2012; Uganda UNPS for years 2009, 2010, 2011, 2013 and 2015. The data has been adjusted for inflation using 2011 as the reference year to reflect price changes over the analysis period.

## 4 Results

### 4.1 Transitional probabilities in and out of CHE

Table 2 displays the transitional probabilities based on the CHE status of Malawi, Tanzania, and Uganda for the three different definitions at 5% and 10% of the budget share in panels A and B respectively, while panel C shows the results for 25% of the capacity-to-pay. The table presents the transitions between adjacent waves with the number of waves varying for each country depending on the data availability.

**Table 2:** Transitional probabilities between different states of catastrophic health expenditures.

Country	No CHE year "t"		CHE year "t"	
	No CHE in year "t+1"	CHE in year "t+1"	No CHE in year "t+1"	CHE in year "t+1"
**Panel A. 5% Total budget share**				
***Wave 1 to wave 2***				
***Malawi (2010 to 2013)***	0.8656 [0.0119]	0.1344 [0.0119]	0.7701 [0.0275]	0.2299 [0.0275]
***Tanzania (2008 to 2010)***	0.8162 [0.0345]	0.1838 [0.0345]	0.6755 [0.0868]	0.3245 [0.0868]
***Uganda (2009 to 2010)***	0.7699 [0.0181]	0.2301 [0.0181]	0.6998 [0.0248]	0.3002 [0.0248]
***Wave 2 to wave 3***				
***Malawi (2013 to 2016)***	0.7216 [0.0156]	0.2784 [0.0156]	0.5876 [0.038]	0.4124 [0.038]
***Tanzania (2010 to 2012)***	0.8482 [0.0342]	0.1518 [0.0342]	0.7001 [0.1025]	0.2999 [0.1025]
***Uganda (2010 to 2011)***	0.7567 [0.018]	0.2433 [0.018]	0.6349 [0.033]	0.3651 [0.033]
***Wave 3 to wave 4***				
***Uganda (2011 to 2013)***	0.8178 [0.0156]	0.1822 [0.0156]	0.6174 [0.0355]	0.3826 [0.0355]
***Wave 4 to wave 5***				
***Uganda (2013 to 2015)***	0.8565 [0.0156]	0.1435 [0.0156]	0.7326 [0.0334]	0.2674 [0.0334]
**Panel B. 10% Total budget share**				
***Wave 1 to wave 2***				
***Malawi (2010 to 2013)***	0.9465 [0.007]	0.0535 [0.007]	0.8331 [0.032]	0.1669 [0.032]
***Tanzania (2008 to 2010)***	0.9206 [0.0223]	0.0794 [0.0223]	0.8698 [0.0352]	0.1302 [0.0352]
***Uganda (2009 to 2010)***	0.8931 [0.0111]	0.1069 [0.0111]	0.8395 [0.0265]	0.1605 [0.0265]
***Wave 2 to wave 3***				
***Malawi (2013 to 2016)***	0.8522 [0.0121]	0.1478 [0.0121]	0.6197 [0.0559]	0.3803 [0.0559]
***Tanzania (2010 to 2012)***	0.9078 [0.0294]	0.0922 [0.0294]	0.914 [0.0389]	0.086 [0.0389]
***Uganda (2010 to 2011)***	0.8491 [0.0145]	0.1509 [0.0145]	0.8169 [0.0357]	0.1831 [0.0357]
***Wave 3 to wave 4***				
***Uganda (2011 to 2013)***	0.8953 [0.0114]	0.1047 [0.0114]	0.7729 [0.0443]	0.2271 [0.0443]
***Wave 4 to wave 5***				
***Uganda (2013 to 2015)***	0.9509 [0.0083]	0.0491 [0.0083]	0.8347 [0.0374]	0.1653 [0.0374]
**Panel C. 25% Capacity to pay**				
***Wave 1 to wave 2***				
***Malawi (2010 to 2013)***	0.9056[0.0099]	0.0944[0.0099]	0.7788[0.0351]	0.2212[0.0351]
***Tanzania (2008 to 2010)***	0.8747[0.0286]	0.1253[0.0286]	0.8103[0.0341]	0.1897[0.0341]
***Uganda (2009 to 2010)***	0.9159[0.0093]	0.0841[0.0093]	0.8635[0.0293]	0.1365[0.0293]
***Wave 2 to wave 3***				
***Malawi (2013 to 2016)***	0.8015[0.0137]	0.1985[0.0137]	0.6673[0.0431]	0.3327[0.0431]
***Tanzania (2010 to 2012)***	0.9098[0.0166]	0.0902[0.0166]	0.6529[0.1382]	0.3471[0.1382]
***Uganda (2010 to 2011)***	0.8657[0.0136]	0.1343[0.0136]	0.8241[0.0416]	0.1759[0.0416]
***Wave 3 to wave 4***				
***Uganda (2011 to 2013)***	0.9347[0.0089]	0.0653[0.0089]	0.8517[0.0411]	0.1483[0.0411]
***Wave 4 to wave 5***				
***Uganda (2013 to 2015)***	0.9789[0.0045]	0.0211[0.0045]	0.8673[0.0402]	0.1327[0.0402]
**Number of households (H)**	**Uganda [1295]**	**Tanzania [2905]**	**Malawi [1661]**	
**Number of waves (T)**	5	**3**	**3**	
**Number of observations (H x T)**	**Uganda [**6475**]**	**Tanzania [8715]**	**Malawi [4983]**	

**Notes:** The estimates are conditional probabilities based on balanced panel using longitudinal weights. Malawi IHPS for years 2010, 2013 and 2016; Tanzania TNSP for years 2008, 2010, and 2012; Uganda UNPS for years 2009, 2010, 2011, 2013 and 2015. The data has been adjusted for inflation using 2011 as the reference year to reflect price changes over the analysis period. CHE defined at 5% and 10% of the budget share are based on Wagstaff and Doorslaer (2003). CHE at 25% of the capacity-to-pay are based on the methodology proposed by Xu et al. (2003).

The possible transitions considered are persistency out of CHE, moving into CHE, moving out of CHE and persistency in CHE.

Table 2 shows that the risk of persistency in CHE between two periods (fourth column) is higher than the risk of moving into CHE (second column), but lower than both moving out of CHE (third column) and persistency in non-CHE (first column) for all three countries across all definitions and thresholds. Moving out of CHE or remaining without CHE are the most likely outcomes. The first column indicates that most households without CHE in one period remain so in the next, with a conditional probability of 75%–95%. Most people with CHE in period t will move out of it in period t+1, with probabilities ranging from 58% to 87% (third column).

The risk of persistency in CHE ranges from 23% to 41% (fourth column). Transition probabilities into CHE range from 13% to 27% (second column).

Generally, the risk of moving into CHE is highest with the 5% threshold of household budget across all countries, lowest at 10% in Malawi and Tanzania, and at 25% of capacity to pay in Uganda. Trends in transitioning into CHE are consistent across definitions in Malawi and Uganda but not in Tanzania. Trends in CHE persistency are consistent across definitions in Malawi and Uganda, similar to transitioning trends. In Tanzania, CHE persistency trends vary by definition; budget share approaches indicate a reduction, while capacity to pay indicates an increase.

Overall, these findings highlight the dynamics of CHE in the examined countries. They suggest that the risk of CHE persistency is lower than the risk of non-CHE persistency but not negligeable. Its magnitude is always sensitive to the CHE definition used unlike its trend over time which can be robust to that.

### 4.2 The mean and counting approach to CHE

**Fig. 2:**
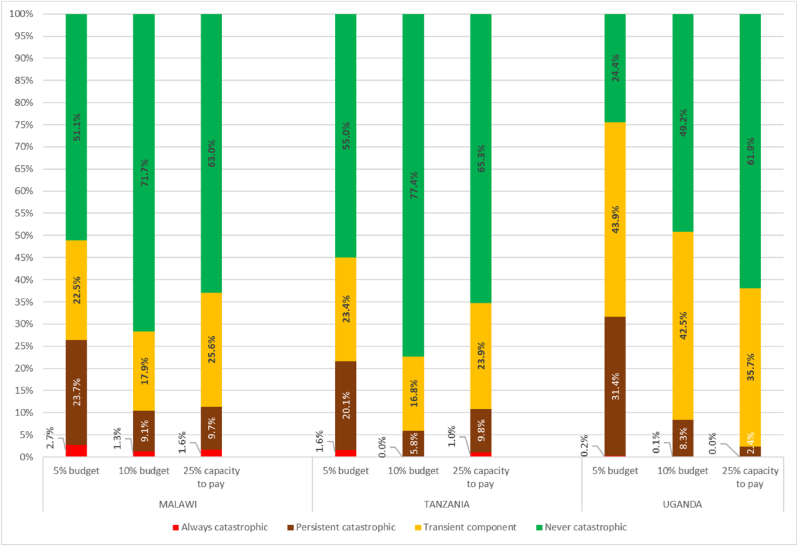
The mean approach applied to various CHE definitions **Notes:** We used balanced panel using longitudinal weights. The statistics are based on Malawi IHPS for years 2010, 2013 and 2016; Tanzania TNSP for years 2008, 2010 and 2012; Uganda UNPS for years 2009, 2010, 2011, 2013 and 2015. The data has been adjusted for inflation using 2011 as the reference year to reflect price changes over the analysis period. We used following definitions of CHE.^***1***^***Always catastrophic*** is CHE at all dates. ***Chronic component*: *persistent*** means time-mean share health expenditures are above τ, but not CHE at all dates. ***Chronic component*: *transient*** means time-mean share health expenditures are not above τ, but with CHE at some dates. ***Never catastrophic*** is no CHE at any date. CHE defined at 5% and 10% of the budget share are based on Wagstaff and Doorslaer (2003). CHE at 25% of the capacity-to-pay are based on the methodology proposed by Xu et al. (2003).

Fig. 2 provides the measures of persistence derived from the mean approach in each country.

In all the definitions, we observe a consistent assessment of persistency in CHE, albeit with substantial differences in the magnitudes depending on the definition used. Most population never encounters CHE, regardless of the thresholds and definitions applied, with the lowest rates at the 5% threshold of household's budget in all three countries and the highest at the 10% in Malawi and Tanzania but at the 25% of household's capacity to pay in Uganda. Following this, transient CHE comes next. It ranges between 17% and 24% in Tanzania, 18% and 26% in Malawi and 36% and 44% in Uganda depending on the definition. It has the lowest rates at the 10% threshold in the two countries with the fewer number of waves (Malawi and Tanzania) and at the 25% threshold of capacity to pay in the country with the longest panel (Uganda). Transient CHE is the highest at 5% of household's budget in Uganda and at 25% of capacity to pay in Malawi and Tanzania. Chronic CHE includes those with persistent and perpetual or “always” CHE. In all the measures we use and all countries, the chronic component of CHE consistently falls below the transient one and it is the lowest of all components. It is particularly low with the capacity to pay definition applied to the longest panel (Uganda with only 2.4% of persistent and 0% of always). But in the panels with 3 waves, the lowest rates are estimated at the 10% threshold of household's budget. Chronic CHE is always the highest with the 5% budget share definition with persistent rates ranging between 20.1% in Tanzania and 31.4% in Uganda and perpetual rates ranging between 0.2% in Uganda and 2.7% in Malawi.

**Fig. 3:**
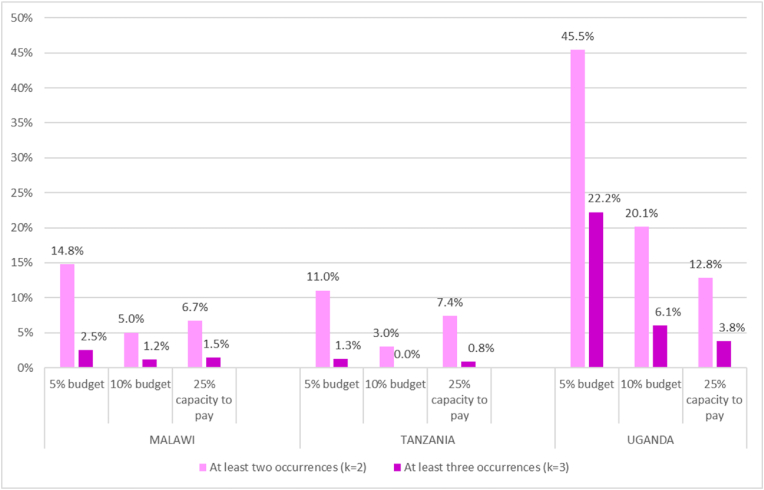
The counting approach applied to various CHE definitions **Note:** We used balanced panel using longitudinal weights. The statistics are based on Malawi IHPS for years 2010, 2013 and 2016; Tanzania TNSP for years 2008, 2010 and 2012; Uganda UNPS for years 2009, 2010, 2011, 2013 and 2015. The data has been adjusted for inflation using 2011 as the reference year to reflect price changes over the analysis period. We used following definitions of CHE. k = 2 households have at least two periods with CHE. k = 3 households have at least three periods with CHE. CHE defined at 5% and 10% of the budget share are based on Wagstaff and Doorslaer (2003). CHE at 25% of the capacity-to-pay are based on the methodology proposed by Xu et al. (2003).

Fig. 3 presents data on the recurrence of CHE across the three countries during the observation period. The recurrence at 5% of the budget share is always higher than with any other definition and for any duration cut-off (at least twice or at least thrice). The recurrence is the lowest for any duration cut-off with the 10% budget share definition in Malawi and Tanzania but with the 25% capacity-to-pay definition in Uganda. Overall Uganda has the highest recurrence of CHE of all three countries for any duration cut-off and Tanzania the lowest recurrence. The estimates obtained through the counting approach are sensitive to the number of observation periods with larger ones increasing the likelihood of finding recurrent CHE at any given intertemporal threshold.

In the Supplementary Material, we present the sample estimates for both mean and counting approaches with (Table 5) and without longitudinal weights (Table 6), and the weighted unbalanced panel tracking all households available in the panel (Table 7), and the results do not change much. The most significant variation in the magnitude of our estimates arises from the different definitions used to determine CHE. When defined in terms of capacity-to-pay with a threshold of 25%, the estimates are smaller compared to those based on 5% of household's budget.

**Fig. 4:**
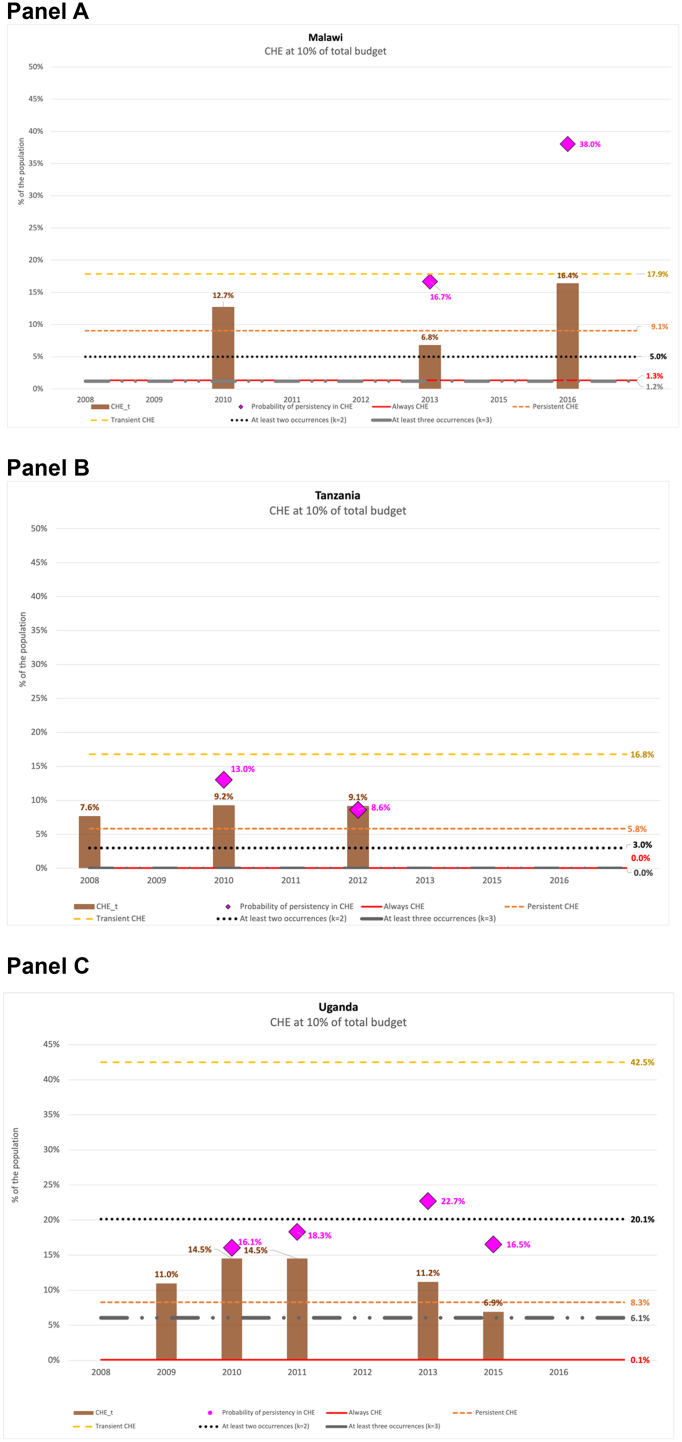
Catastrophic Health Expenditure: cross-sectional rates vs transient, chronic, persistent, and recurrent rates **Note:** We used balanced panel using longitudinal weights. The statistics are based on Malawi IHPS for years 2010, 2013 and 2016; Tanzania TNSP for years 2008, 2010 and 2012; Uganda UNPS for years 2009, 2010, 2011, 2013 and 2015. The data has been adjusted for inflation using 2011 as the reference year to reflect price changes over the analysis period. We used following definitions of CHE. k = 2 households have at least two periods with CHE. k = 3 households have at least three periods with CHE. CHE defined at 5% of the budget share is based on Wagstaff and Doorslaer (2003). Figures based on other definitions are included in the Supplementary material.

Fig. 4 visually complements the standard cross-sectional measures of CHE with the three approaches proposed in this paper, using the 5% budget share definition. Similar figures based on other definitions are available in the supplemental materials. The brown bars show the standard CHE rate at each point in time, in Malawi (panel A), Tanzania (panel B), and Uganda (panel C). The pink dots indicate the short-term probability of persistency in CHE between two waves, while the other lines show rates of transient (yellow), chronic (red and orange), and recurrent (gray and black) CHE.

The three approaches reveal important aspects of CHE. First, the short-term probability of persistency in CHE is generally higher than the cross-sectional CHE rate, particularly in shorter panels like Malawi and Tanzania, and in most periods in Uganda. Second, the trend in cross-sectional CHE rates does not always match the short-term persistency trends (brown bars versus pink dots). For example, in Uganda (2009–2010), the CHE rate dropped by 10 percentage points, but persistency risk in the short-term increased by 6.5 points. In Tanzania (2010–2012), the CHE rate remained the same, but the persistency risk decreased by 2.5 points.

Third, even when trends align, the magnitudes differ. In Malawi (2013–2016), the CHE rate rose by 14 percentage points, while persistency increased by 18.2 points. In Uganda (2011–2013), the CHE rate fell by 2 points, but persistency dropped by 11.6 points.

Fourth, short-term persistency is typically higher than long-term persistency (pink dots versus red and orange dashed lines). All these points (first to fourth) are robust to the CHE definition (see Figs. 1 and 2 in supplemental materials). At the 5% budget definition of CHE the long-term persistency rates lie between cross-sectional rates (brown bar versus orange dashed line). For instance, in Uganda, cross-sectional CHE rates range from 17% to 34%, long-term persistency is 31.4%, and short-term persistency is 26.7%–38.3%. But this results is not robust to the CHE definition.

Finally, the rate of the population always facing CHE is very small, decreasing with longer panels (red lines in panel C versus panel A and B in Fig. 4 and figures in the supplemental materials). Overall, these findings show that cross-sectional changes underestimate the risk of persistency in the short-term for a significant fraction of the population. In the three countries analyzed, at least 27% of those facing CHE are likely to experience it again in the next wave at the 5% budget definition. But the cross-sectional rate is in general higher than the rate experiencing chronic CHE or recurrent CHE. However, none of these long-term measures is negligeable.

Table 3 present the marginal effects of our empirical models for various approaches for CHE at 5% of budget share. Horizontally, each country panel is divided into four columns. In the leftmost column, labeled, “CHE-first Differences.”, the dependent variable in the pooled OLS model presented in equation (1) corresponds to the difference in CHE outcomes between period “t+1” and period “t” described in Table 2. Zero indicates no change in CHE states, 1 corresponds to transitioning into CHE and −1 indicates moving out of CHE. The regression models aim to identify the factors associated with the dynamics in CHE in the three countries in columns (1), (5), and (9), rather than cross-sectional prevalence.

**Table 3:** Regression results for persistent catastrophic measures.

Variables	UGANDA				TANZANIA				MALAWI			
	(1)	(2)	(3)	(4)	(5)	(6)	(7)	(8)	(9)	(10)	(11)	(12)
	Δin**CHE**	**Transient**	**Persistent**	**Chronic k=2**	Δin**CHE**	**Transient**	**Persistent**	**Chronic k=2**	Δin**CHE**	**Transient**	**Persistent**	**Chronic k=2**
	β[SE]	β[SE]	β[SE]	β[SE]	β[SE]	β[SE]	β[SE]	β[SE]	β[SE]	β[SE]	β[SE]	β[SE]
**Household characteristics**												
***Urban/Rural(=1)***	−0.0177	0.08*	−0.09**	−0.11**	−0.0033	0.00	−0.09**	−0.05*	−0.0087	−0.05*	−0.06**	−0.03
	[0.0184]	[0.035]	[0.029]	[0.033]	[0.0153]	[0.039]	[0.026]	[0.023]	[0.0249]	[0.026]	[0.021]	[0.019]
***Household owned(=1)***	0.16*	0.03	−0.01	0.01	0.04	−0.03	−0.08*	−0.06*	–	–	–	–
	[0.080]	[0.026]	[0.023]	[0.025]	[0.064]	[0.040]	[0.035]	[0.027]	–	–	–	–
***Household size***	−0.0035	0.01*	−0.01+	−0.00	−0.0074*	0.00	−0.01+	0.00	0.01	0.00	−0.00	0.01+
	[0.0032]	[0.004]	[0.004]	[0.004]	[0.0034]	[0.006]	[0.004]	[0.003]	[0.018]	[0.004]	[0.003]	[0.003]
***HH head is over 60 years(=1)***	0.0097	−0.01	0.00	0.01	0.0258	−0.57**	0.04	0.13**	−0.0806	−0.03	−0.08+	−0.04
	[0.0198]	[0.030]	[0.028]	[0.029]	[0.0673]	[0.148]	[0.091]	[0.039]	[0.0524]	[0.049]	[0.043]	[0.037]
***HH head is female(=1)***	−0.0113	0.04	0.03	0.06+	0.0071	0.06	−0.02	−0.11**	0.0127	−0.01	−0.01	0.04*
	[0.0202]	[0.029]	[0.028]	[0.029]	[0.0242]	[0.043]	[0.044]	[0.037]	[0.0237]	[0.021]	[0.025]	[0.019]
**Education of HH head (**No education – reference)												
***Primary education(=1)***	−0.0138	0.00	0.09**	0.08**	−0.0377*	−0.02	−0.00	−0.01	−0.0717	0.05	−0.02	−0.06+
	[0.0194]	[0.028]	[0.026]	[0.027]	[0.0176]	[0.034]	[0.032]	[0.025]	[0.0497]	[0.035]	[0.041]	[0.035]
***Secondary education(=1)***	−0.0186	0.12**	−0.01	0.04	−0.0301	0.00	−0.03	−0.03	–	0.04	−0.12**	−0.08*
	[0.0246]	[0.039]	[0.033]	[0.037]	[0.0241]	[0.057]	[0.042]	[0.040]	–	[0.038]	[0.043]	[0.037]
***University or equivalent(=1)***	0.0230	0.12*	−0.06	−0.02	0.0851	0.34**	−0.23**	−0.14**	–	−0.01	−0.03	−0.15**
	[0.0303]	[0.055]	[0.044]	[0.050]	[0.0686]	[0.121]	[0.061]	[0.048]	–	[0.052]	[0.056]	[0.040]
Marital status of the Household head (Monogamous-reference)												
***Married polygamous(=1)***	0.0287	0.03	−0.14**	−0.15**	0.0035	0.09+	−0.02	0.06+	−0.0359	−0.03	0.04	0.07*
	[0.0190]	[0.029]	[0.025]	[0.027]	[0.0215]	[0.046]	[0.039]	[0.034]	[0.1500]	[0.037]	[0.035]	[0.035]
***Living together(=1)***	–	–	–	–	–	0.04	−0.11**	−0.03	–	–	–	–
	–	–	–	–	–	[0.034]	[0.025]	[0.022]	–	–	–	–
***Separated(=1)***	–	–	–	–	–	0.08	−0.08+	−0.11**	–	–	–	–
	–	–	–	–	–	[0.065]	[0.049]	[0.034]	–	–	–	–
***Divorced(=1)***	0.0256	−0.00	−0.03	−0.04	0.0095	0.06	−0.01	0.19*	0.0807	−0.04+	0.06*	−0.01
	[0.0282]	[0.046]	[0.044]	[0.046]	[0.0290]	[0.086]	[0.095]	[0.091]	[0.0947]	[0.024]	[0.028]	[0.021]
***Never Married(=1)***	0.0108	−0.17*	−0.13+	−0.17*	−0.0263	0.22+	0.04	−0.12**	0.0563	–	–	–
	[0.0547]	[0.073]	[0.077]	[0.078]	[0.0400]	[0.122]	[0.128]	[0.031]	[0.1766]	–	–	–
***Widower(=1)***	0.0040	−0.02	−0.03	−0.01	0.0401	0.10+	0.03	−0.02	0.0810	–	–	–
	[0.0265]	[0.038]	[0.035]	[0.037]	[0.0303]	[0.060]	[0.056]	[0.048]	[0.0970]	–	–	–
**Employment situation Household head**												
***Hours worked per week***	−0.0003	−0.05*	0.02	−0.01						−0.07*	−0.08**	−0.02
	[0.0004]	[0.024]	[0.022]	[0.024]	–	–	–	–	–	[0.027]	[0.025]	[0.025]
***Employment status(=1)***	−0.0073	–	–	–	0.0539	−0.49**	0.10	0.16**	−0.0071	–	–	–
	[0.0215]	–	–	–	[0.0628]	[0.134]	[0.079]	[0.030]	[0.0210]	–	–	–
**Members age in years**												
Children between 0 and 5	−0.0021	0.00	0.02	0.04**	−0.0107	0.02	−0.01	−0.01	−0.0050	−0.03*	0.03**	−0.04**
	[0.0062]	[0.013]	[0.013]	[0.014]	[0.0067]	[0.018]	[0.012]	[0.010]	[0.0106]	[0.013]	[0.011]	[0.011]
Household members aged 60 or more	0.0029	−0.00	0.02*	0.02+	0.0043	0.03	0.02	−0.01	0.0665	0.03	0.05	0.03
	[0.0060]	[0.009]	[0.009]	[0.009]	[0.0193]	[0.044]	[0.034]	[0.023]	[0.0356]	[0.033]	[0.029]	[0.026]
**Health care use and health shock**												
Have used hospital care***(=1)***	0.2611***	0.02	0.15**	0.19**	0.0775***	0.06*	0.08**	0.19**	0.1649***	0.03	0.18**	0.12**
	[0.0190]	[0.027]	[0.027]	[0.026]	[0.0107]	[0.032]	[0.026]	[0.025]	[0.0363]	[0.031]	[0.032]	[0.031]
Have used outpatient care***(=1)***	0.2808***	−0.10	0.32**	0.22*	0.0820***	0.01	0.16**	0.07**	0.3840***	0.05**	0.25**	0.17**
	[0.0479]	[0.070]	[0.083]	[0.085]	[0.0070]	[0.028]	[0.023]	[0.019]	[0.0254]	[0.021]	[0.022]	[0.020]
Household head health shocks ***(=1)***	0.0074	−0.03	0.06*	0.06*	0.0600*	−0.07	−0.01	0.05	−0.0185	−0.00	0.02	0.02
	[0.0142]	[0.028]	[0.028]	[0.029]	[0.0275]	[0.052]	[0.039]	[0.036]	[0.0241]	[0.023]	[0.019]	[0.019]
Constant	−0.0450	0.31**	0.31**	0.38**	−0.0324	0.65**	0.18*	0.04	−0.0065	0.20**	0.18**	0.14**
	[0.0308]	[0.043]	[0.040]	[0.042]	[0.0658]	[0.147]	[0.089]	[0.044]	[0.0050]	[0.038]	[0.044]	[0.036]
**Observations**	**6505**	**3610**	**3610**	**3610**	**5810**	**2601**	**2601**	**2601**	**4983**	**4983**	**4983**	**4983**
**R-squared**	**0.03576**	**0.027**	**0.061**	**0.066**	**0.04032**	**0.037**	**0.095**	**0.094**	**0.11191**	**0.019**	**0.148**	**0.102**

**Notes:** Balanced panel using longitudinal IPWT weights. Malawi IHPS for years 2010, 2013 and 2016; Tanzania TNSP for years 2008, 2010 and 2012; Uganda UNPS for years 2009, 2010, 2011, 2013 and 2015. Balanced panels using longitudinal IPWT weights. The data has been adjusted for inflation using 2011 as the reference year to reflect price changes over the analysis period. CHE defined at 5% and 10% of the budget share are based on Wagstaff and Doorslaer (2003). CHE at 25% of the capacity-to-pay are based on the methodology proposed by Xu et al. (2003). ***Persistent*** means time-mean share health expenditures are above τ, but not CHE at all dates. T***ransient*** means time-mean share health expenditures are not above τ, but with CHE at some dates. Chronic k = 2 households have at least two periods with CHE.

Household head education generally reduces the likelihood of transitioning into CHE. In Tanzania, heads with primary education are 3.7% less likely to face CHE than those with no education. In Uganda, owning a house increases the probability of CHE, while in Tanzania, larger households reduce this probability. However, these effects are not statistically significant.

Hospitalizations, seeking healthcare, or health shocks significantly increase CHE likelihood. Specifically, hospitalization increases the likelihood by 26% in Uganda (column1) and 16% in Malawi (column 9). Seeking health care raises the likelihood by 38% in Malawi (column 9), Uganda by 26% (column1), and by 8% (column5) in Tanzania. Health shocks increase CHE likelihood by 6% in Tanzania.

First differences in CHE provides only ideas about the period by period transitions and does not take into account the longer time periods. To examine longer time spans, Table 3 shows the marginal effects of the pooled OLS model applied to the outcomes of the mean approach (Fig. 2) in the columns labeled “TRANSIENT” (2), (6) and (10) and “PERSISTENT” (3), (7) and (11). The marginal effects for the double occurrence of CHE illustrated in Fig. 3 are presented in the columns titled “CHRONIC,t = 2” (4), (8) and (12).

We find that specific socioeconomic, demographic, and housing characteristics impact the duration of CHE differently under the 5% budget share definition. In Tanzania, an increase in the household head's age is associated with a 57% decrease in the probability of facing transient CHE (column 6), while the same age increase is positively linked to chronic OOP health spending (column 8). The impact of being a female household head varies, as it reduces the likelihood of recurrent CHE in Tanzania but increases it in Malawi (column 12).

The presence of children under five years of age correlates with chronic, recurrent CHE in Uganda (column 4) and persistent CHE in Malawi (column 11). Interestingly, in Malawi, the presence of children reduces the likelihood of experiencing only transient CHE outcomes. Conversely, in Uganda, the presence of older adults significantly increases the likelihood of persistent CHE. Across all countries, larger household sizes are consistently associated with both persistent and recurrent CHE, highlighting a common trend irrespective of specific national contexts.

Higher education levels among household heads substantially reduce the likelihood of recurrent (columns 8 and 12) and persistent (column 7) CHE in Tanzania and Malawi. However, they increase the probability of facing transient CHE in Uganda and Tanzania (columns 2 and 6). Having a secondary degree reduces both the likelihood of persistent and recurrent CHE in Malawi (columns 11 and 12). Conversely, when household heads have only attained primary school education, they are more likely to face chronic and persistent CHE in Uganda (columns 3 and 4).

Additionally, we find several sociodemographic correlates of chronic and persistent CHE. In Tanzania and Malawi, households with divorced or separated heads are more likely to face chronic (column 8) and persistent (column 11) CHE than monogamous households. For polygamous households, the results are mixed: they increase the probability of chronic CHE in Malawi (column 12) but decrease the probability of persistent (column 3) and recurrent (column 4) CHE in Uganda when compared to monogamous households.

While we couldn't identify households with employment-tied insurance, we found that working longer hours is associated with a lower probability of facing transient CHE in Uganda and Malawi (columns 2 and 10). Conversely, being in full-time employment is associated with the recurrence of CHE in Tanzania.

Hospitalizations, seeking health care, and facing health shocks in households are significantly and positively associated with all duration metrics of CHE for all the panels we analyzed. Similarly, households in urban areas are less likely to face persistent CHE in all countries (columns (3), (7), and (11)) and chronic in the case of Tanzania (column (8)) and Uganda (column (4)). We attribute this trend to the concentration of healthcare access and health insurance coverage in urban settings.

## 5 Discussion and conclusion

In this study, we propose three methods to assess the persistency of CHE. This is important as even small but persistent OOP spending can accumulate to catastrophic lifetime costs (French and Jones, 2004; Nardi et al., 2017). We apply the methods to panel data from Malawi, Uganda, and Tanzania with different durations and length. To assess the robustness of findings, we use three different CHE definitions (5% and 10% of the budget share and 25% of the capacity to pay). The 10% budget share corresponds to SDG indicator 3.8.2. Higher threslholds tend to concentrate CHE among the wealthy, masking effects on poorer households. But lower OOP spending can affect poor or near-poor households significantly. Thresholds of 5% for total consumption and 25% for non-food consumption are widely used, as they capture these impacts better (Doorslaer et al., 2007).

We found a fair degree of mobility by assessing two points in time and over extended periods, through transitional probabilities, changes in relation to mean CHE, and CHE recurrence. Across the three countries, we find that at least 27% of the people facing CHE in one period because they spent more than 5% of their household budget on health out-of-pocket, will face it again in the next period. The lower-bound short-term risk decreases with other definitions, but it is not negligeable. Comparing the three different approaches applied to the three CHE definitions, we consistently find that the short-term probability of persistency in CHE is generally higher than the cross-sectional CHE rate, particularly in shorter panels like Malawi and Tanzania, and in most periods in Uganda. The trend in cross-sectional CHE rates does not always match the short-term persistency trends; even when trends align, the magnitudes differ; the short-term persistency is typically higher than long-term persistency and the rate of the population always facing CHE is very small, decreasing with longer panels. These findings highlight the need for policy interventions promoting affordable healthcare.

In the panels we analyzed, between 11% and 45% of the population incurred CHE at least twice during the observation period when using the 5% budget definition of CHE. The double recurrence rate ranges between 7% and 13% when using the 25% capacity to pay definition and between 3% and 20% when using the 10% budget definition. Between 22% and 32% of the population experienced chronic CHE at the 5% budget definition (6%–10% at the 10% budget definition of CHE; 2%–11% at 25% of capacity to pay). Persistent CHE may reflect deficiencies in the health system, such as limited coverage, shortages of doctors or medicines, or the need to seek private services due to long waiting times not only during health emergencies but also in cases of chronic diseases and longer treatments.

Our panel datasets do not distinguish between types of illness, such as acute or chronic. However, factors like an aging population and lifestyle choices contribute significantly to the rise in chronic disease burden globally. Thus, the age composition of household heads and members is a relevant proxy for non-communicable diseases (Miranda et al., 2008; Sun and Li, 2023). Su et al. (2004) found that while professional care-seeking was similar for children and older adults, illness episodes among older adults were significantly associated with CHE. We found similar results, with a significant association between persistent and recurrent CHE among households with elderly members in Uganda and Malawi. The elderly population, a growing group in the region (Mavrodaris et al., 2013), is more likely to experience chronic diseases that increase healthcare expenditure (Su et al., 2004). This highlights the importance of targeted support for the aging population, including healthcare subsidies, social support programs, and pension schemes, to ensure their financial stability and protect them from healthcare costs.

Education and gender are relevant key determinants for persistent CHE. The study indicates that households with female heads are more susceptible to chronic CHE in certain countries. As for education, higher levels of education are associated with a higher likelihood of experiencing transient CHE and a lower probability of incurring persistent and recurring CHE. This underscores the advantages of improved education and better access to financial protection mechanisms for healthcare. To address this disparity, gender-specific policies that empower women economically and provide targeted support for female-headed households can help alleviate their vulnerability to CHE. Additionally, interventions that promote gender equality in education and employment could contribute to reducing the financial burden on households led by women.

Seeking health care and, in particular, hospitalizations are strongly significant across all metrics we use to assess intertemporal CHE. Previous studies have indicated that hospitalization expenses are the highest expenditure item in terms of money spent on healthcare (Attia-Konan et al., 2019). Our findings indicate that the financial stress related to hospitalization is strongly associated with recurrent and persistent outcomes, suggesting long-lasting effects that compromise future household expenditures. (Okoroh and Riviello, 2021) reported gaps in the availability and affordability of medicines and anesthesia, emphasizing the need to improve procurement practices to bolster investments in health and healthcare services (Mekuria and Ali, 2023). For context, it's worth noting that high-income countries allocate approximately 10% of their GDP to health, whereas low-income countries allocate less than 5% (Ekman, 2007).

It's important to note the limitations of our study. The panels we used were primarily designed for agricultural purposes, providing valuable socioeconomic information but limited data on health-related characteristics like healthcare access and utilization. This information is relevant for vulnerable populations, that our study might overlook, as previous studies have shown that household incomes play a crucial role in determining out-of-pocket health expenditures and are negatively associated with forgoing healthcare. (Ssozi and Amlani, 2015). In the sub-Saharan region, many low-income insured households opt to forgo seeking health care due to the financial challenges associated with OOP health payments (You et al., 2020).

Additionally, panel durations varied across countries, with Uganda having five waves and Malawi and Tanzania had only three, which restricted our ability to fully explore the long-term effects of health shocks on household welfare. The discrepancies in panel lengths also affect the comparability of metrics like recurrence and mean changes over time. While we were able to use balanced panels of the same length in Malawi and Tanzania, Uganda's data has unevenly distributed collection points over its five waves. Additionally, recall periods for OOP varied within the panels, potentially introducing memory bias.

Despite these limitations, our findings can provide a foundational framework for more in-depth discussions and analyses concerning the challenges posed by OOP in Sub-Saharan Africa. These insights can provide information for the development of targeted strategies to alleviate the detrimental impacts of such expenses on households and communities in the region.

## Ethics approval and consent to participate

No formal ethical approval is required because all the analysis is done on existing online data with open access.

## Ethics approval and consent to participate

The analysis was only based on secondary published data. The panel data protocols were submitted for approval to the Ethics, Biosecurity and Research Committees of the National Institute of Public Health for all countries we analyzed. We confirm that all methods were carried out in accordance with relevant guidelines and regulations in the declaration with ethics approval and consent to participate section. The data we use is anonymized before its use.

## CRediT authorship contribution statement

**Rocio Garcia-Diaz:** Writing – review & editing, Writing – original draft, Supervision, Project administration, Methodology, Formal analysis, Data curation, Conceptualization. **Vishnu Prasad Sapkota:** Methodology, Data curation, Formal analysis. **Gabriela Flores:** Writing – review & editing, Supervision, Methodology, Investigation, Formal analysis, Conceptualization.

## Data Availability

Data will be made available on request.
