# Supplementary material for: Persistency of catastrophic out-of-pocket health expenditures: Measurement with evidence from three African countries - Malawi, Tanzania, and Uganda
Source: Soc Sci Med. 2024 Sep;357:117156. doi: 10.1016/j.socscimed.2024.117156 (PMC11413478; doi:10.1016/j.socscimed.2024.117156)
Supplement: Multimedia component 1 [file mmc1.docx]

# **Supplementary material**

***Uganda***

We use five waves of data from the Uganda National Panel Survey (UNPS). The data were collected by the Uganda Bureau of Statistics with support from the World Bank. The UNPS aimed to track and interview a nationally representative sample of 2,926 households interviewed (2009-2010). Each household was interviewed twice a year, following a household questionnaire that collected information on households' socio-demographics such as education, health, work, income, and expenditure. the sample was 2,926 for wave 1 (2009-2010), followed by four subsequent waves: Wave 2 (2010-2011), wave 3 (2011-2012), wave 4 (2013-2014), and wave 5 (2015-2016). The UNPS carries the longest period in our panel data study, and it also has the highest attrition rate in our pool of countries, accounting for 53% in wave 5. In the Appendix, Table 1, we present all panel information with attrition rates and the use of refreshment data, new randomly sampled households that are given the questionnaire at the same time in the follow-up waves in the panels. The total balanced sample size is 1,295 observations for each wave.

***Tanzania***

We use three waves of data from the Tanzania National Panel Survey (TNPS) implemented by the National Bureau of Statistics of the United Republic of Tanzania for the years, 2008, 2010, and 2012. The TNPS survey followed a multistage, stratified, random sampling to generate nationally representative samples. The initial sample was 3265 households. The questionnaire provides information on household characteristics and basic facilities, education, health, and income and expenditure. 98% of the initial sample was re-interviewed in wave 2 (2010-2011) and 94% in wave 3 (2012-2013). Table 1 shows that the attrition rate together with availability of complete information on our variables of interest till the final collection survey accounts for 11% concerning the baseline sample in wave 1. We retained a balanced sample of 2905 households followed in all subsequent waves and contained complete information on our variables of interest in each wave, which forms 89% of initial sample at baseline.

***Malawi***

We use three waves of the Malawi Integrated Household Survey (IHPS) which was conducted by Malawi's National Statistical Office. The initial sample was drawn from a stratified and randomly selected sample of households from which a subsample of households was included in the Agricultural Input Support Survey, representing 2,032 households in wave 1 (2010-2011). The questionnaires cover information about housing infrastructure, information on household members' education, health, labor force participation, consumption, and expenditure. The two other rounds were conducted in 2013-2014 (wave 2) and 2016-2017 (wave 3) and they are presented in the appendix in Table A.1. A total of 1,661 households were tracked across the three waves in the panel, which forms 88% of the initial sample.

Selective attrition is a concern that arises in longitudinal studies. The attrition rate tends to vary significantly across different panels, generally increasing as the panel lengthens. For instance, in Uganda's UNPS, the third wave experienced an attrition rate of 24.5% compared to the baseline year, while by the sixth wave in 2015, the attrition rate reached 53.26%. On the other hand, in the third wave of both panels for Malawi and Tanzania, the attrition rates were much lower at 6.8% and 6%, respectively.

| Table 1. Panel structure of the three countries covered in the analysis. | | | | | | | |
| --- | --- | --- | --- | --- | --- | --- | --- |
| **Year** | **Base year observations** | **Followed up from base year** | **New observations** | **Drop-out observations** | **Household followed in all periods** | **Attrition** | **Total observations** |
| **Uganda** | | | | | | | |
| **2009** | 2560 |  |  |  | 1295 |  | 2560 |
| **2010** |  | 2352 | 302 | 763 | 1295 | 24.49% | 2654 |
| **2011** |  | 2339 | 483 | 776 | 1295 | 24.91% | 2822 |
| **2013** |  | 1563 | 267 | 1552 | 1295 | 49.82% | 3116 |
| **2015** |  | 1456 | 247 | 1659 | 1295 | 53.26% | 3294 |
| **Tanzania** | | | | | | | |
| **2008** | 3265 |  |  |  | 2905 |  | 3265 |
| **2010** |  | 3166 | 0 | 99 | 2905 | 3.03% | 3864 |
| **2012** |  | 3039 | 0 | 92 | 2905 | 4.01% | 5010 |
| **Malawi** | | | | | | | |
| **2010** | 2032 |  |  |  | 1661 |  | 2032 |
| **2013** |  | 1721 | 311 | 310 | 1661 | 15.30% | 1721 |
| **2016** |  | 1908 | 124 | 105 | 1661 | 6.10% | 1908 |
| **Notes:** The statistics are based on the Uganda Panel Survey a longitudinal panel survey (UNPS) of 2009, 2010, 2011, 2013 and 2015; The Malawi Integrated Household Survey (IHHS) of 2010, 2013 and 2016. The Tanzania National Panel Survey (TNPS) of 2008, 2010 and 2012. | | | | | | | |

| **Attrition treatment and selection bias**  To limit the potential bias resulting from attrition, we use inverse probability weights (IPW) to correct for the probability bias resulting from observable characteristics in the sample (Fitzgerald, Gottschalk, and Moffitt, 1998; Wooldridge, 2003). This procedure entails utilizing baseline characteristics (household head’s gender, education, household size and composition, household's ownership, geolocation of the households), time-varying characteristics (household head’s move in the last 5 years or migration status), and fixed effects on the years, to predict the likelihood of individuals dropping out of the sample. Consequently, while cross-sectional weights (*hhw*) adjust the composition of observations for a specific period, longitudinal weights (*lhhw*) derived from the IPW perform the same adjustment across all waves in the panel as further explained hereafter.  The IPW uses the predicted probability as a weight in the subsequent waves in the analysis. The IPW is a weight equal to 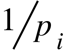, where 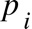 represents the probability of dropping out of the sample. By doing so, more weight is assigned to individuals whose observable characteristics indicate a higher probability of attrition in the sample (or regressions). In the subsequent analysis, we utilize the longitudinal weights (lhhw), obtained by multiplying the cross-sectional hhw by 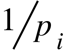 . Table A.2 in the appendix shows the logistic regression results to estimate the IPW for all panels included in the analysis. The results presented in this study use weighted balanced sample estimates across all three panels. However, in the Table 2, results are provided for sample estimates and unbalanced weighted panels for all three countries.  Table 2: Estimated model results for IPWT computations for three countries | | | |
| --- | --- | --- | --- |
| **Dependent variable** | **Logistic model for loss of follow-up (=1)** | | |
| **Covariates** | **UGANDA** | **MALAWI** | **TANZANIA** |
| Household head is male | 0.35**[0.035] | -0.12[0.111] | 0.03**[0.057] |
| Age of household head | 0.01**[0.001] | 0.02**[0.004] | 0.04**[0.003] |
| Household size | 0.09**[0.011] | -0.09**[0.032] | 0.09**[0.022] |
| Annual household Income | <0.0001[<0.001] | <0.0001[<0.0001] | <-0.0001[<0.0001] |
| Household owned | -0.25**[0.039] |  | 0.62**[0.057] |
| Urban | -0.3400[0.041] | -0.13[0.093] | 0.05[0.055] |
| Total number of males in household | -0.04**[0.009] | 0.06[0.044] | 0.03[0.027] |
| Number of adults >60 years-old | -0.12**[0.016] | -0.13[0.110] | -0.54**[0.057] |
| Head move in the last five years | -0.56**[0.071] |  |  |
| Children between 0-5 years old | -0.08**[0.017] | 0.21**[0.049] | -0.02[0.030] |
| Young adults between 20-24 years old | -0.16**[0.025] | -0.18**[0.056] | -0.39**[0.034] |
| 2010 year | -0.16**[0.057] |  | -1.00**[0.073] |
| 2011 year | 0.02 [0.075] |  |  |
| 2012 year |  |  | -1.77**[0.069] |
| 2013 year | -0.16**[0.059] | 1.88**[0.145] |  |
| 2015 year | 1.01 [0.067] |  |  |
| 2016 year |  | 0.34**[0.096] |  |
| **Observations** | **14971** | **5100** | **11983** |
| Notes: The statistics are based on the Uganda Panel Survey a longitudinal panel survey (UNPS) of 2009, 2010, 2011, 2013 and 2015; The Malawi Integrated Household Survey (IHHS) of 2010, 2013 and 2016. The Tanzania National Panel Survey (TNPS) of 2008, 2010 and 2012. **p<0.01, *p<0.05, +<0.10. Estimated coefficients are log-odds. Standard errors in brackets. | | | |

| Table 3: Descriptive statistics of the household in each wave for the three countries. | | | | | | | | | | | | | | | | | | | | | | | | | | | | | | | | | | | | | | | |
| --- | --- | --- | --- | --- | --- | --- | --- | --- | --- | --- | --- | --- | --- | --- | --- | --- | --- | --- | --- | --- | --- | --- | --- | --- | --- | --- | --- | --- | --- | --- | --- | --- | --- | --- | --- | --- | --- | --- | --- |
| **Variables** | **UGANDA** | | | | | | | | | | | | | | | | **TANZANIA** | | | | | | | | | | | | **MALAWI** | | | | | | | | | | |
|  | **2009** | | **2010** | | **2011** | | | | **2013** | | | | **2015** | | | | **2008** | | | | **2010** | | | | **2012** | | | | **2010** | | | | **2013** | | | | **2016** | | |
|  | **Mean** | **SD** | **Mean** | **SD** | **Mean** | | **SD** | | **Mean** | | **SD** | | **Mean** | | **SD** | | **Mean** | | **SD** | | **Mean** | | **SD** | | **Mean** | | **SD** | | **Mean** | | **SD** | | **Mean** | | **SD** | | **Mean** | | **SD** |
| **OOP spending (LCU/d)** | 579 | 1274 | 537 | 1947 | 609 | | 1959 | | 671 | | 2095 | | 580 | | 2822 | | 252 | | 967 | | 425 | | 2066 | | 668 | | 9822 | | 13 | | 90 | | 20 | | 49 | | 78 | | 174 |
| **Age of household head** | 45.2 | 15.3 | 46.35 | 15.54 | 46.19 | | 15.14 | | 48.63 | | 15.03 | | 48.07 | | 15.19 | | 45.76 | | 1532 | | 46.14 | | 15.72 | | 47.73 | | 15.62 | | 41.11 | | 15.91 | | 43.8 | | 15.29 | | 47.72 | | 15.08 |
| **Household head is female** | 0.11 | 0.47 | 0.38 | 0.48 | 0.39 | | 0.49 | | 0.39 | | 0.49 | | 0.43 | | 0.5 | | 0.25 | | 0.43 | | 0.25 | | 0.43 | | 26 | | 0.44 | | 0.21 | | 0.41 | | 0.23 | | 0.42 | | 0.31 | | 0.46 |
| **Education of HH head** |  |  |  |  |  | |  | |  | |  | |  | |  | |  | |  | |  | |  | |  | |  | |  | |  | |  | |  | |  | |  |
| No education (=1) | 0.19 | 0.19 | 0.16 | 0.36 | 0.18 | | 0.19 | | 0.19 | | 0.39 | | 0.16 | | 0.36 | | 0.23 | | 42 | | 0.23 | | 0.42 | | 0.22 | | 0.41 | | 0.05 | | 0.22 | | 0.05 | | 0.22 | | 0.05 | | 0.21 |
| Primary education (=1) | 0.51 | 0.5 | 0.53 | 0.5 | 0.52 | | 0.5 | | 0.55 | | 0.5 | | 0.51 | | 0.5 | | 0.61 | | 0.49 | | 0.6 | | 0.49 | | 0.61 | | 0.49 | | 0.57 | | 0.5 | | 0.57 | | 0.5 | | 0.57 | | 0.5 |
| Secondary Education (=1) | 0.2 | 0.4 | 0.22 | 0.41 | 0.22 | | 0.41 | | 0.21 | | 0.4 | | 22 | | 0.42 | | 0.15 | | 36 | | 0.16 | | 0.37 | | 0.16 | | 0.37 | | 33 | | 0.47 | | 0.32 | | 0.47 | | 32 | | 0.47 |
| Higher Education (=1) | 0.09 | 0.29 | 0.1 | 0.3 | 0.08 | | 0.27 | | 0.09 | | 0.29 | | 0.11 | | 0.31 | | 0.01 | | 0.1 | | 0.01 | | 0.1 | | 0.02 | | 0.12 | | 0.06 | | 0.24 | | 0.06 | | 0.25 | | 0.06 | | 0.24 |
| **Marital state of the HH head** |  |  |  |  |  |  | |  | |  | |  | |  | |  | |  | |  | |  | |  | |  | |  | |  | |  | |  | |  | |  |  |
| Monogamous (=1) | 0.54 | 0.5 | 0.53 | 0.5 | 0.55 | | 0.5 | | 0.54 | | 0.5 | | 0.54 | | 0.5 | | 0.52 | | 0.5 | | 0.46 | | 0.5 | | 0.51 | | 0.5 | | 0.72 | | 0.45 | | 0.73 | | 0.44 | | 0.66 | | 0.47 |
| Polygamous (=1) | 0.18 | 39 | 0.18 | 0.19 | 0.2 | | 0.4 | | 0.17 | | 0.38 | | 0.18 | | 0.39 | | 0.13 | | 33 | | 0.11 | | 0.31 | | 0.11 | | 0.32 | | 0 | | 0.25 | | 0 | | 0.23 | | 7 | | 0.25 |
| Living together (=1) |  | - | - | - | - | | - | | - | |  | | - | | - | | 0.08 | | 28 | | 0.15 | | 0.36 | | 0.09 | | 0.29 | |  | | - | | - | | - | | - | | - |
| Separated (=1) |  | - | - | - | - | | - | | - | |  | | - | | - | | 0.07 | | 0.26 | | 0.07 | | 0.26 | | 0.07 | | 0.25 | |  | | - | | - | | - | | - | | - |
| Divorced (=1) | 0.1 | 0.1 | 0.1 | 0.3 | 0.1 | | 0.29 | | 0.11 | | 0.31 | | 0.09 | | 0.29 | | 0.02 | | 0.15 | | 0.03 | | 0.17 | | 0.04 | | 0.20 | | - | | - | | - | | - | | - | |  |
| Widower (=1) | 0.14 | 35 | 0.15 | 0.35 | 0.13 | | 0.33 | | 0.16 | | 0.37 | | 16 | | 0.37 | | 0.13 | | 33 | | 0.12 | | 0.31 | | 0.14 | | 0.34 | | - | | - | | - | | - | | - | |  |
| Never Married (=1) | 0.04 | 0.19 | 0.04 | 0.19 | 0.01 | | 0.16 | | 0.02 | | 0.14 | | 0.02 | | 0.15 | | 0.05 | | 0.21 | | 0.05 | | 0.23 | | 0.05 | | 0.21 | | 0.22 | | 0.41 | | 0.21 | | 0.41 | | 0.28 | | 0.45 |
| **Hours worked pc week** | 29.03 | 23:20 | 29.03 | 21.7 | 30.97 | | 22.33 | | 32.18 | | 21.46 | | 30.62 | | 21.11 | | - | |  | | - | | - | | - | | - | | - | | - | | 12.15 | | 24.82 | | 1318 | | 26.42 |
| **Employment states** |  | - | - | - | - | | - | | - | |  | | - | | - | | 0.76 | | 0.43 | | 0.97 | | 0.16 | | 0.98 | | - | |  | | - | | - | | - | | - | | - |
| **No. of children: 0-4** | 0.91 | 0.97 | 0.99 | 1.02 | 0.99 | | 1 | | 0.83 | | 0.9 | | 0.53 | | 0.71 | | 0.8 | | 0.92 | | 0.78 | | 0.95 | | 0.79 | | 1 | | 0.88 | | 0.84 | | 0.88 | | 0.85 | | 0.94 | | 0.96 |
| **Members aged 60 or more** | 0.63 | 0.17 | 0.98 | 1.54 | 1.51 | | 2.14 | | 0.33 | | 0.59 | | 0.68 | | 1.17 | | 0.31 | | 0.59 | | 0.32 | | 0.6 | | 35 | | 0.62 | | 0.22 | | 0.51 | | 0.24 | | 0.53 | | 0.11 | | 0.61 |
| **Hospital care (=1)** | 0.23 | 0.42 | 0.14 | 0.34 | 0.12 | | 0.33 | | 0.15 | | 0.36 | | 0.10 | | 0.30 | | 0.22 | | 0.23 | | 0.42 | | 0.38 | | 0.19 | | 0.39 | | 0.06 | | 0.25 | | 0.07 | | 0.26 | | 0.12 | | 0.32 |
| **Outpatient care (=1)** | 0.03 | 0.17 | 0.01 | 0.1 | 0.02 | | 0.12 | | 0.04 | | 0.2 | | 0.01 | | 0.1 | | 0.41 | | 0.49 | | 0.44 | | 0.5 | | 38 | | 0.49 | | 0.16 | | 0.36 | | 0.15 | | 0.36 | | 30 | | 0.46 |
| **Health shocks** | 0.19 | 0.4 | 0.24 | 0.43 | 0.15 | | 36 | | 0.13 | | 0.33 | | 0.1 | | 0.3 | | 0.11 | | 0.31 | | 0.08 | | 0.28 | | 0.07 | | 0.26 | | 0.23 | | 0.42 | | 0.22 | | 0.42 | | 28 | | 0.45 |
| **Urban (=1)** | 0.28 | 0.45 | 0.24 | 0.43 | 9.21 | | 0.41 | | 0.26 | | 0.44 | | 0.24 | | 0.43 | | 0.36 | | 0.48 | | 0.34 | | 0.47 | | 35 | | 0.48 | | 0.73 | | 0.66 | | 0.72 | | 0.55 | | 0.74 | | 0.66 |
| **Household owned house (=1)** | 0.77 | 0.42 | 0.82 | 0.39 | 0.81 | | 39 | | 0.84 | | 0.37 | | 0 | | 0 | | 0.78 | | 0.42 | | 0.72 | | 0.45 | | 0.75 | | 0.44 | | - | |  | | - | | - | | - | | - |
| **HH size** | 6.19 | 3.41 | 7.01 | 3.65 | 7.59 | | 1.80 | | 5.93 | | 2.96 | | 5.73 | | 3.01 | | 5.18 | | 2.9 | | 5.23 | | 3.1 | | 533 | | 3.11 | | 4.51 | | 2.37 | | 4.53 | | 2.1 | | 5.37 | | 2.32 |
| **Number of households** | **1295** | | | | | | | | | | | | | | | | **2905** | | | | | | | | | | | | **1661** | | | | | | | | | | |

Notes: Notes: The statistics are based on the balanced panel of Uganda Panel Survey a longitudinal panel survey (UNPS) of 2009, 2010, 2011, 2013 and 2015; The Malawi Integrated Household Survey (IHHS) of 2010, 2013 and 2016. The Tanzania National Panel Survey (TNPS) of 2008, 2010 and 2012. longitudinal weights are applied to produce the estimates.

| Table 4: Catastrophic health expenditure in each wave of the countries using the three definitions | | | | | | | |
| --- | --- | --- | --- | --- | --- | --- | --- |
| **Country** | **Year** | **Catastrophic Health Expenditure (CHE) defined at** | | | | | |
|  |  | **5% of the budget share** | | **10% of the budget share** | | **25% of the capacity-to-pay** | |
|  |  | **Mean** | **SE** | **Mean** | **SE** | **Mean** | **SE** |
| Malawi | 2010 | 0.2237 | [0.0131] | 0.1268 | [0.0104] | 0.1334 | [0.0107] |
| Malawi | 2013 | 0.1562 | [0.0112] | 0.0678 | [0.0074] | 0.1118 | [0.0099] |
| Malawi | 2016 | 0.3000 | [0.0145] | 0.1637 | [0.0120] | 0.2154 | [0.0132] |
| Tanzania | 2008 | 0.2018 | [0.0103] | 0.0764 | [0.0066] | 0.1455 | [0.0090] |
| Tanzania | 2010 | 0.2094 | [0.0099] | 0.0924 | [0.0070] | 0.1413 | [0.0084] |
| Tanzania | 2012 | 0.2092 | [0.0101] | 0.0913 | [0.0071] | 0.1558 | [0.0090] |
| Uganda | 2009 | 0.3428 | [0.0154] | 0.1751 | [0.0121] | 0.1207 | [0.0104] |
| Uganda | 2010 | 0.2375 | [0.0148] | 0.1096 | [0.0100] | 0.0822 | [0.0083] |
| Uganda | 2011 | 0.2546 | [0.0179] | 0.1451 | [0.0165] | 0.1156 | [0.0108] |
| Uganda | 2013 | 0.2293 | [0.0133] | 0.1116 | [0.0098] | 0.0734 | [0.0081] |
| Uganda | 2015 | 0.1691 | [0.0126] | 0.0691 | [0.0088] | 0.0342 | [0.0057] |
| Notes: The statistics are based on the balanced panel sample size for each country reported in Table 1 in Annex. All the estimates are weighted using longitudinal weights. SE are available in brackets. CHE defined at 5% and 10% of the budget share are based on Wagstaff and Doorslaer (2003). CHE at 10% corresponds to sustainable development indicator 3.8.2. CHE at 25% of the capacity-to-pay are based on the methodology proposed by Xu et al. (2003) | | | | | | | |

| \| Table 5: Persistency of CHE – estimates using mean and counting approach with longitudinal weights \| \| \| \| \| \| \| \| \| \| \| --- \| --- \| --- \| --- \| --- \| --- \| --- \| --- \| --- \| --- \| \|  \| **MALAWI** \| \| \| **TANZANIA** \| \| \| **UGANDA** \| \| \| \| **Denominator:** \| **Budget share** \| \| **CTP** \| **Budget share** \| \| **CTP** \| **Budget share** \| \| **CTP** \| \| **Threshold(s):** \| **5%** \| **10%** \| **25%** \| **5%** \| **10%** \| **25%** \| **5%** \| **10%** \| **25%** \| \| **Panel A: Mean approach^1^** \|  \|  \|  \|  \|  \|  \|  \|  \|  \| \| **Never catastrophic** \| 0.5113 \| 0.7173 \| 0.6299 \| 0.5498 \| 0.7735 \| 0.6527 \| 0.2445 \| 0.4916 \| 0.6193 \| \| [0.0089] \| [0.0080] \| [0.0086] \| [0.0082] \| [0.0070] \| [0.0079] \| [0.0065] \| [0.0075] \| [0.0072] \| \| **Transient component** \| 0.2252 \| 0.1787 \| 0.2565 \| 0.234 \| 0.1678 \| 0.2392 \| 0.4392 \| 0.4251 \| 0.3569 \| \| [0.0075] \| [0.0069] \| [0.0079] \| [0.0070] \| [0.0063] \| [0.0071] \| [0.0074] \| [0.0073] \| [0.0070] \| \| **Chronic component** \|  \|  \|  \|  \|  \|  \|  \|  \|  \| \| ***Always catastrophic*** \| 0.0267 \| 0.0134 \| 0.0163 \| 0.0155 \| 0.0005 \| 0.0104 \| 0.0021 \| 0.0007 \| 0 \| \| [0.0030] \| [0.0020] \| [0.0022] \| [0.0018] \| [0.0002] \| [0.0015] \| [0.0006] \| [0.0003] \| - \| \| ***Persistent catastrophic*** \| 0.2368 \| 0.0905 \| 0.0973 \| 0.2007 \| 0.0582 \| 0.0977 \| 0.3143 \| 0.0827 \| 0.0239 \| \| [0.0076] \| [0.0050] \| [0.0052] \| [0.0067] \| [0.0038] \| [0.0049] \| [0.0068] \| [0.0040] \| [0.0024] \| \| **Panel B: Counting approach^2^** \|  \|  \|  \|  \|  \|  \|  \|  \|  \| \| $k=2$ \| 0.1479 \| 0.0501 \| 0.0673 \| 0.1104 \| 0.0299 \| 0.074 \| 0.4545 \| 0.2012 \| 0.1282 \| \| [0.0059] \| [0.0036] \| [0.0040] \| [0.0088] \| [0.0049] \| [0.0076] \| [0.0073] \| [0.0060] \| [0.0048] \| \| $k=3$ \| 0.0249 \| 0.0119 \| 0.0149 \| 0.0128 \| 0 \| 0.0085 \| 0.2223 \| 0.0605 \| 0.038 \| \| [0.0027] \| [0.0018] \| [0.0019] \| [0.0028] \| - \| [0.0024] \| [0.0061] \| [0.0036] \| [0.0028] \| \| **Total households (H)** \| **1661** \| \| \| **2905** \| \| \| **1295** \| \| \| \| **Number of waves (T)** \| **3** \| \| \| **3** \| \| \| **5** \| \| \| \| **Total observations (H X T)** \| **4983** \| \| \| **8715** \| \| \| **6475** \| \| \| \| **Note:** CTP refers to the capacity to pay. Balanced panel using longitudinal weights. Malawi IHPS for years 2010, 2013 and 2016; Tanzania TNSP for years 2008, 2010 and 2012; Uganda UNPS for years 2009, 2010, 2011, 2013 and 2015The data has been adjusted for inflation using 2011 as the reference year to reflect price changes over the analysis period. We used following definitions of CHE.  ***^1^Always catastrophic*** is catastrophic health payments at all dates.  ***Chronic component*: *persistent*** means time-mean share health expenditures are above $\tau$, but not CHE at all dates.  ***Chronic component*: *transient*** means time-mean share health expenditures are not above $\tau$, but with CHE at some dates.  ***Never catastrophic*** is no CHE at any date.  ^2^$k$=2 at least two periods with CHE.  $k$=3 at least three periods with CHE.  CHE defined at 5% and 10% of the budget share are based on Wagstaff and Doorslaer (2003). CHE at 10% corresponds to sustainable development indicator 3.8.2. CHE at 25% of the capacity-to-pay are based on the methodology proposed by Xu et al. (2003) \| \| \| \| \| \| \| \| \| \|   Table 6: CHE estimates using mean and counting methodology using balanced samples without weights | | | | | | | | | |
| --- | --- | --- | --- | --- | --- | --- | --- | --- | --- | --- | --- | --- | --- | --- | --- | --- | --- | --- | --- | --- | --- | --- | --- | --- | --- | --- | --- | --- | --- | --- | --- | --- | --- | --- | --- | --- | --- | --- | --- | --- | --- | --- | --- | --- | --- | --- | --- | --- | --- | --- | --- | --- | --- | --- | --- | --- | --- | --- | --- | --- | --- | --- | --- | --- | --- | --- | --- | --- | --- | --- | --- | --- | --- | --- | --- | --- | --- | --- | --- | --- | --- | --- | --- | --- | --- | --- | --- | --- | --- | --- | --- | --- | --- | --- | --- | --- | --- | --- | --- | --- | --- | --- | --- | --- | --- | --- | --- | --- | --- | --- | --- | --- | --- | --- | --- | --- | --- | --- | --- | --- | --- | --- | --- | --- | --- | --- | --- | --- | --- | --- | --- | --- | --- | --- | --- | --- | --- | --- | --- | --- | --- | --- | --- | --- | --- | --- | --- | --- | --- | --- | --- | --- | --- | --- | --- | --- | --- | --- | --- | --- | --- | --- | --- | --- | --- | --- | --- | --- | --- | --- | --- | --- | --- | --- | --- | --- | --- | --- | --- | --- | --- | --- | --- | --- | --- | --- | --- | --- | --- | --- | --- | --- | --- | --- | --- | --- | --- | --- | --- | --- | --- | --- | --- | --- | --- | --- | --- | --- | --- | --- | --- | --- | --- | --- | --- | --- | --- | --- | --- | --- | --- | --- | --- | --- | --- | --- | --- | --- | --- | --- | --- | --- | --- |
|  | **MALAWI** | | | **TANZANIA** | | | **UGANDA** | | |
| **Denominator:** | **Budget share** | | **CTP** | **Budget share** | | **CTP** | **Budget share** | | **CTP** |
| **Threshold(s):** | **5%** | **10%** | **25%** | **5%** | **10%** | **25%** | **5%** | **10%** | **25%** |
| **Panel A: Mean approach^1^** |  |  |  |  |  |  |  |  |  |
| **Never catastrophic** | 0.5523 | 0.7500 | 0.6715 | 0.5711 | 0.7831 | 0.6885 | 0.2398 | 0.4881 | 0.6118 |
|  | [0.0131] | [0.0114] | [0.0123] | [0.0092] | [0.0076] | [0.0086] | [0.0118] | [0.0139] | [0.0135] |
| **Transient component** | 0.2156 | 0.1570 | 0.2266 | 0.2220 | 0.1556 | 0.2172 | 0.4381 | 0.4274 | 0.3643 |
|  | [0.0108] | [0.0096] | [0.0110] | [0.0077] | [0.0067] | [0.0077] | [0.0138] | [0.0137] | [0.0133] |
| **Chronic component** |  |  |  |  |  |  |  |  |  |
| ***Always catastrophic*** | 0.0207 | 0.0103 | 0.0138 | 0.0151 | 0.0007 | 0.0086 | 0.0023 | 0.0008 | 0.0000 |
|  | [0.0037] | [0.0027] | [0.0031] | [0.0023] | [0.0005] | [0.0017] | [0.0013] | [0.0008] | [0.0000] |
| ***Persistent catastrophic*** | 0.2114 | 0.0826 | 0.0882 | 0.1917 | 0.0606 | 0.0857 | 0.3198 | 0.0838 | 0.0238 |
|  | [0.0107] | [0.0072] | [0.0074] | [0.0073] | [0.0044] | [0.0052] | [0.0129] | [0.0077] | [0.0042] |
| **Panel B: Counting approach^2^** |  |  |  |  |  |  |  |  |  |
| $k=2$ | 0.1294 | 0.0433 | 0.0602 | 0.1038 | 0.0274 | 0.0582 | 0.4505 | 0.1964 | 0.1282 |
|  | [0.0082] | [0.0050] | [0.0058] | [0.0103] | [0.0055] | [0.0079] | [0.0136] | [0.0109] | [0.0092] |
| $k=3$ | 0.0193 | 0.0090 | 0.0126 | 0.0148 | 0.0000 | 0.0068 | 0.2211 | 0.0615 | 0.0375 |
|  | [0.0034] | [0.0023] | [0.0027] | [0.0041] | [0.0000] | [0.0028] | [0.0114] | [0.0066] | [0.0052] |
| **Total households (H)** | **1661** | | | **2905** | | | **1295** | | |
| **Total waves (T)** | **3** | | | **3** | | | **5** | | |
| **Total observations (H X T)** | **4983** | | | **8715** | | | **6475** | | |
| **Note:** CTP refers to the capacity to pay. Balanced panel using longitudinal IPWT weights. Malawi IHPS for years 2010, 2013 and 2016; Tanzania TNSP for years 2008, 2010, and 2012; Uganda UNPS for years 2009, 2010, 2011, 2013 and 2015.  ***Always catastrophic*** is catastrophic health payments at all dates.  ***Chronic component*: *persistent*** means time-mean share health expenditures are above $\tau$, but not CHE at all dates.  ***Chronic component*: *transient*** means time-mean share health expenditures are not above $\tau$, but with CHE at some dates.  ***Never catastrophic*** is no CHE at any date.  ^2^$k$=2 at least two periods with CHE.  $k$=3 at least three periods with CHE.  CHE defined at 5% and 10% of the budget share are based on Wagstaff and Doorslaer (2003). CHE at 10% corresponds to sustainable development indicator 3.8.2. CHE at 25% of the capacity-to-pay are based on the methodology proposed by Xu et al. (2003) | | | | | | | | | |

.

| Table 7: CHE estimates using mean and counting methodology using unbalanced samples | | | | | | | | | |
| --- | --- | --- | --- | --- | --- | --- | --- | --- | --- |
|  | **MALAWI** | | | **TANZANIA** | | | **UGANDA** | | |
| **Denominator:** | **Budget share** | | **CTP** | **Budget share** | | **CTP** | **Budget share** | | **CTP** |
| **Threshold(s):** | **5%** | **10%** | **25%** | **5%** | **10%** | **25%** | **5%** | **10%** | **25%** |
| **Panel A: Mean approach^1^** |  |  |  |  |  |  |  |  |  |
| **Never catastrophic** | 0.5403 | 0.7252 | 0.6718 | 0.5564 | 0.7781 | 0.6595 | 0.3204 | 0.5447 | 0.6472 |
|  | [0.145] | [0.0138] | [0.0127] | [0.0078] | [0.0066] | [0.0075] | [0.0076] | [0.0074] | [0.0070] |
| **Transient component** | 0.1970 | 0.1746 | 0.2194 | 0.2416 | 0.1693 | 0.2433 | 0.3585 | 0.3676 | 0.3140 |
|  | [0.0088] | [0.0124] | [0.0099] | [0.0069] | [0.0061] | [0.0069] | [0.0069] | [0.0070] | [0.0066] |
| **Chronic component** |  |  |  |  |  |  |  |  |  |
| ***Always catastrophic*** | 0.0270 | 0.0122 | 0.0148 | 0.0103 | 0.0009 | 0.0069 | 0.0060 | 0.0006 | 0.0006 |
|  | [0.0027] | [0.0018] | [0.0019] | [0.0010] | [0.0003] | [0.0008] | [0.0009] | [0.0002] | [0.0003] |
| ***Persistent catastrophic*** | 0.2357 | 0.0880 | 0.0940 | 0.1916 | 0.0518 | 0.0903 | 0.3151 | 0.0871 | 0.0382 |
|  | [0.0138] | [0.0086] | [0.0092] | [0.0073] | [0.0044] | [0.0052] | [0.0129] | [0.0077] | [0.0042] |
| **Panel B: Counting approach^2^** |  |  |  |  |  |  |  |  |  |
| *k*=2 | 0.0373 | 0.1219 | 0.0518 | 0.1159 | 0.0273 | 0.0717 | 0.3696 | 0.1634 | 0.1085 |
|  | [0.0041] | [0.0082] | [0.0044] | [0.0050] | [0.0027] | [0.0043] | [0.0070] | [0.0051] | [0.0044] |
| *k*=3 | 0.0074 | 0.0155 | 0.0093 | 0.0147 | 0.0005 | 0.0099 | 0.1624 | 0.0476 | 0.0309 |
|  | [0.0012] | [0.0017] | [0.0012] | [0.0017] | [0.0002] | [0.0015] | [0.0050] | [0.0033] | [0.0030] |
| **Total observations**  (Households x waves) | **5597** | | | **9734** | | | **13297** | | |
| **Note:** CTP refers to the capacity to pay. Malawi IHPS for years 2010, 2013 and 2016; Tanzania TNSP for years 2008, 2010, and 2012; Uganda UNPS for years 2009, 2010, 2011, 2013 and 2015.  ***Always catastrophic*** is catastrophic health payments at all dates.  ***Chronic component*: *persistent*** means time-mean share health expenditures are above $\tau$, but not CHE at all dates.  ***Chronic component*: *transient*** means time-mean share health expenditures are not above $\tau$, but with CHE at some dates.  ***Never catastrophic*** is no CHE at any date.  ^2^$k$=2 at least two periods with CHE.  $k$=3 at least three periods with CHE.  CHE defined at 5% and 10% of the budget share are based on Wagstaff and Doorslaer (2003). CHE at 10% corresponds to sustainable development indicator 3.8.2. CHE at 25% of the capacity-to-pay are based on the methodology proposed by Xu et al. (2003) | | | | | | | | | |

**Figure 1**. Catastrophic Health Expenditure at 10% of household budget: cross-sectional rates vs transient, chronic, persistent, and recurrent rates

**Panel A**

**
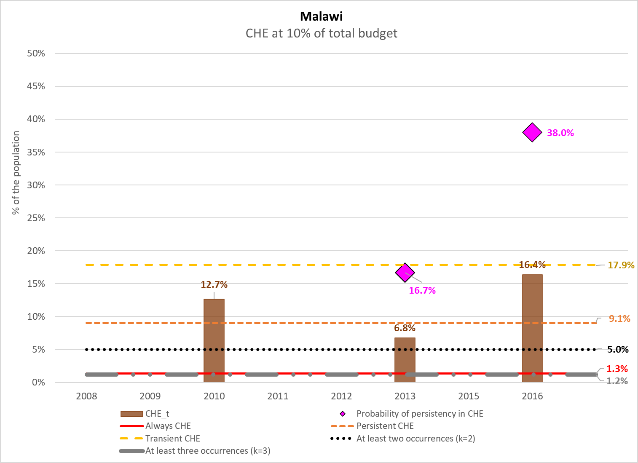
**

**Panel B**

**
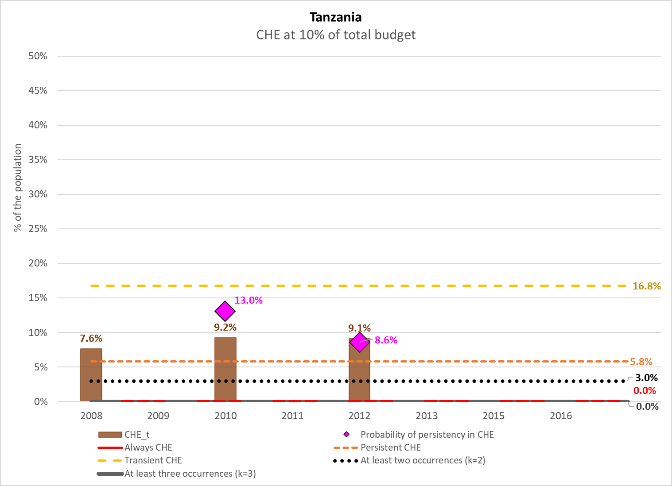
**

**Panel C**

**
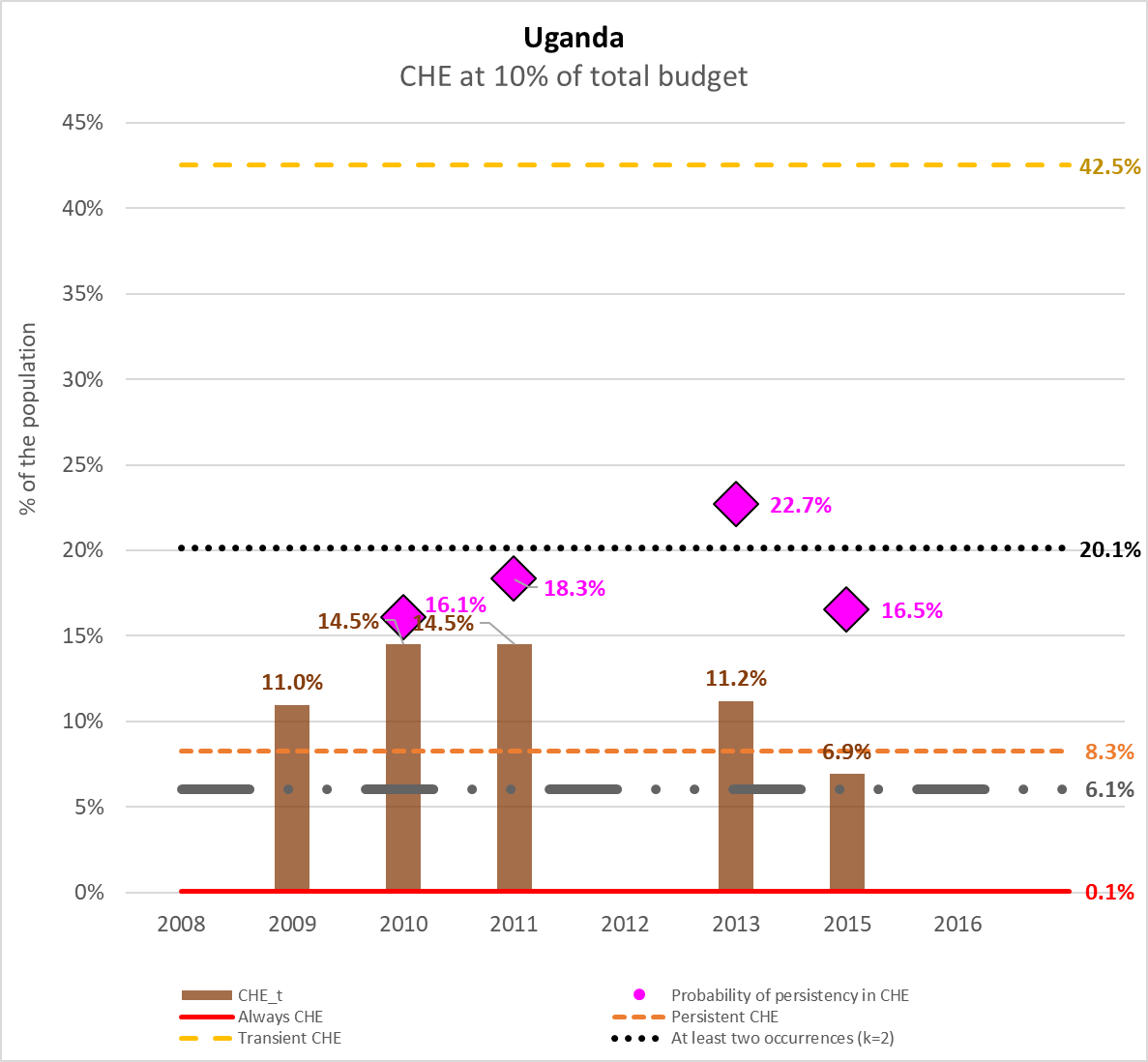
**

**Note:** We used balanced panel using longitudinal weights. The statistics are based on Malawi IHPS for years 2010, 2013 and 2016; Tanzania TNSP for years 2008, 2010 and 2012; Uganda UNPS for years 2009, 2010, 2011, 2013 and 2015. The data has been adjusted for inflation using 2011 as the reference year to reflect price changes over the analysis period. We used following definitions of CHE.

$k$=2 households have at least two periods with CHE.

$k$=3 households have at least three periods with CHE.

CHE defined at 10% of the budget share is based on Wagstaff and Doorslaer (2003). It corresponds to sustainable development indicator 3.8.2.

**Figure 2**. Catastrophic Health Expenditure at 25% of household capacity to pay: cross-sectional rates vs transient, chronic, persistent, and recurrent rates

**Panel A**

**
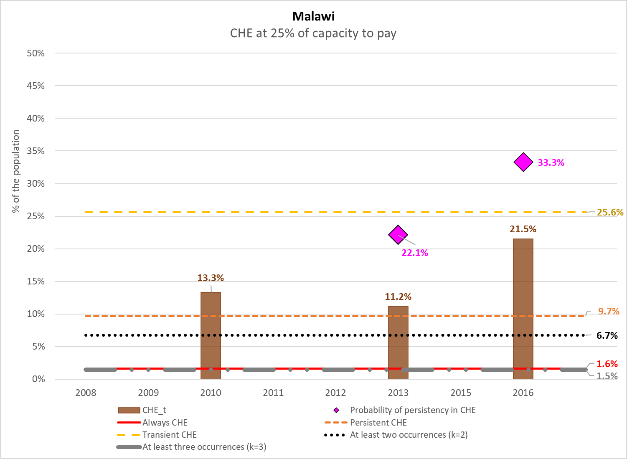
**

**Panel B**

**
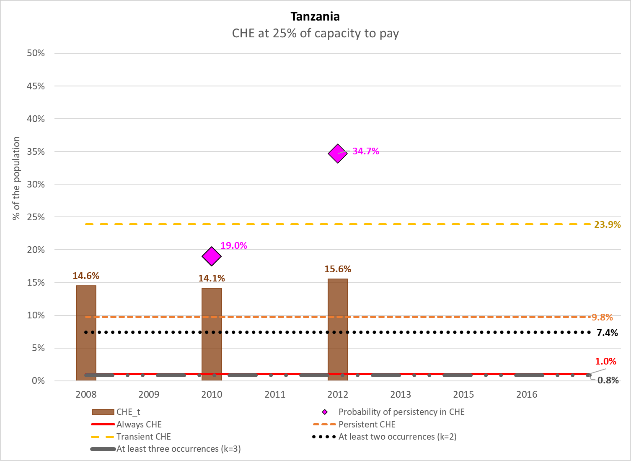
**

**Panel C**

**
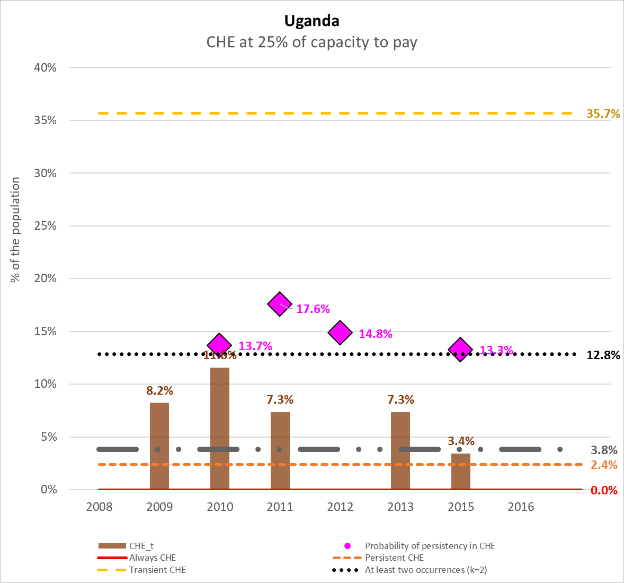
**

**Note:** We used balanced panel using longitudinal weights. The statistics are based on Malawi IHPS for years 2010, 2013 and 2016; Tanzania TNSP for years 2008, 2010 and 2012; Uganda UNPS for years 2009, 2010, 2011, 2013 and 2015. The data has been adjusted for inflation using 2011 as the reference year to reflect price changes over the analysis period. We used following definitions of CHE.

$k$=2 households have at least two periods with CHE.

$k$=3 households have at least three periods with CHE.

CHE at 25% of the capacity-to-pay are based on the methodology proposed by Xu et al. (2003)
